# Molecular profiling of single circulating tumor cells with diagnostic intention

**DOI:** 10.15252/emmm.201404033

**Published:** 2014-10-30

**Authors:** Bernhard Polzer, Gianni Medoro, Sophie Pasch, Francesca Fontana, Laura Zorzino, Aurelia Pestka, Ulrich Andergassen, Franziska Meier-Stiegen, Zbigniew T Czyz, Barbara Alberter, Steffi Treitschke, Thomas Schamberger, Maximilian Sergio, Giulia Bregola, Anna Doffini, Stefano Gianni, Alex Calanca, Giulio Signorini, Chiara Bolognesi, Arndt Hartmann, Peter A Fasching, Maria T Sandri, Brigitte Rack, Tanja Fehm, Giuseppe Giorgini, Nicolò Manaresi, Christoph A Klein

**Affiliations:** 1 Project Group “Personalized Tumor Therapy”, Fraunhofer Institute for Toxicology und Experimental MedicineRegensburg, Germany; 2 Silicon Biosystems S.p.A.Bologna, Italy; 3 Experimental Medicine and Therapy Research, University of RegensburgRegensburg, Germany; 4 Division of Laboratory Medicine, European Institute of OncologyMilan, Italy; 5 Department of Gynecology and Obstetrics, University MunichMunich, Germany; 6 Department of Gynecology and Obstetrics, University of DüsseldorfDüsseldorf, Germany; 7 Department of Pathology, University ErlangenErlangen, Germany; 8 Department of Gynecology and Obstetrics, University ErlangenErlangen, Germany

**Keywords:** breast cancer, circulating tumor cells, metastasis, single cell analysis

## Abstract

Several hundred clinical trials currently explore the role of circulating tumor cell (CTC) analysis for therapy decisions, but assays are lacking for comprehensive molecular characterization of CTCs with diagnostic precision. We therefore combined a workflow for enrichment and isolation of pure CTCs with a non-random whole genome amplification method for single cells and applied it to 510 single CTCs and 189 leukocytes of 66 CTC-positive breast cancer patients. We defined a genome integrity index (GII) to identify single cells suited for molecular characterization by different molecular assays, such as diagnostic profiling of point mutations, gene amplifications and whole genomes of single cells. The reliability of > 90% for successful molecular analysis of high-quality clinical samples selected by the GII enabled assessing the molecular heterogeneity of single CTCs of metastatic breast cancer patients. We readily identified genomic disparity of potentially high relevance between primary tumors and CTCs. Microheterogeneity analysis among individual CTCs uncovered pre-existing cells resistant to *ERBB2*-targeted therapies suggesting ongoing microevolution at late-stage disease whose exploration may provide essential information for personalized treatment decisions and shed light into mechanisms of acquired drug resistance.

## Introduction

Cancer is an evolutionary process where differences in microenvironmental selection pressures, iatrogenic intervention and dynamic cellular changes over time constantly generate variant subpopulations among systemically spread cancer cells (Greaves & Maley, 2012; Klein, 2013). The need to monitor these changes is growing from the recent re-appreciation of the heterogeneity among tumor cells (Gerlinger *et al*, 2012) and the great promise of targeted therapies. Such therapies exploit specific molecular characteristics of the cancer cells (Luo *et al*, 2009); however, heritable genetic and epigenetic changes as well as phenotypic plasticity of the cancer cells often result in acquired drug resistance. Moreover, prolonged disease courses as a consequence of several lines of treatment inevitably lead to the evolution of cancer cells that are increasingly disparate from the primary tumor that was surgically removed long time ago. Hence, primary tumors become less relevant sources of molecular information about systemically spread cancer cells and monitoring cancer evolution over disease courses would be important. However, this is seldom performed. The major reason for this is that repeated intralesional bioptic sampling is rarely tolerable for the patients and often not feasible. Therefore, many clinical studies currently explore the use of circulating tumor cells (CTCs) as a “liquid biopsy”.

Recent technologies for the enrichment and detection of CTCs have shown an association of CTC counts with clinical outcome in breast (Cristofanilli *et al*, 2004; Liu *et al*, 2009; Bidard *et al*, 2014) and other cancers (Danila *et al*, 2007; Cohen *et al*, 2008). Here, the FDA-cleared Cell Search® system (Veridex LLC) has become the gold standard (Cristofanilli *et al*, 2005; de Bono *et al*, 2008; Cohen *et al*, 2008). As metastatic tissue is often inaccessible and substantial heterogeneity has been shown between both the primary tumor and its metastases as well as between different metastases of an individual patient (Stoecklein & Klein, 2010; Almendro *et al*, 2014), molecular CTC analysis could serve as an easily accessible liquid biopsy for metastatic disease. Additionally, repeated sampling and molecular analysis of CTCs could help to uncover traits of cancer cells selected under therapy early on and could enable physicians to rapidly adapt treatment strategies.

However, two major obstacles have limited the exploitation of CTC analysis, first the isolation of individual tumor cells to purity without contaminating white blood cells (WBCs) and second, the ability to comprehensively analyze single cell genomes or phenotypes for diagnostic purposes. We addressed these two problems by combining a novel cell-sorting microsystem based on dielectrophoresis (Manaresi *et al*, 2003) with a deterministic single cell amplification method (Klein *et al*, 1999). The DEPArray™ technology (Silicon Biosystems SpA) enables automated isolation of pure single cells of rare subpopulations within an enriched single cell suspension. After isolation, we performed whole genome amplification (WGA) using the *Ampli*1™ WGA kit, which globally amplifies the genome after generation of defined DNA fragments and adaptor ligation using a single primer to the adaptor sequence.

With these three commercially available methods, we explored the determinants of single CTC analysis and define quality criteria for the diagnostic assessment of HER2 amplifications, PIK3CA mutations and genomic copy number changes.

## Results

### Detection, isolation and whole genome amplification of CTCs

**Figure 1:**
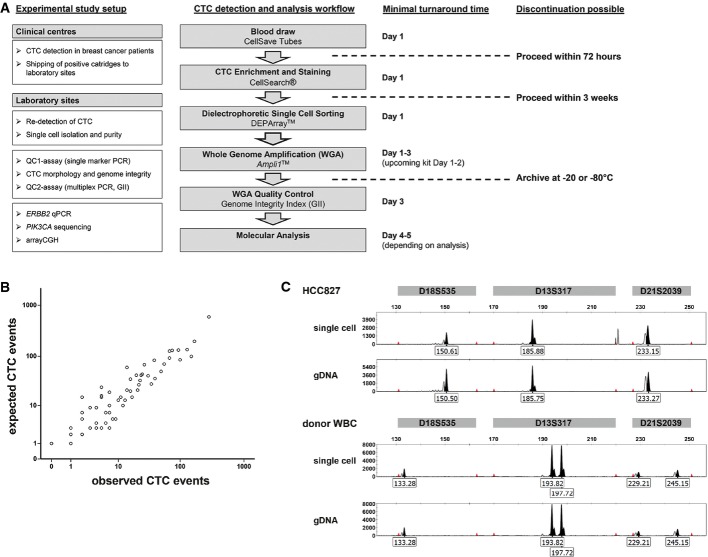
Detection, isolation and purity of single breast cancer CTCs Flowchart summarizing the workflow for single CTC detection and molecular analysis. The boxes on the left depict analyses performed and methods developed during the course of the study. The final workflow is shown in the middle column, and minimal turnaround time for the complete workflow is shown on the right (assuming the blood drawn in the morning of day 1). Dashed lines indicate possible points of discontinuation during the workflow protocol.Correlation between expected (as calculated from CellSearch® CTC count) and observed (as per DEPArray™ system) number of CTCs from 79 samples of 66 breast cancer patients (Spearman's rho correlation coefficient *r* = 0.94, *P* < 0.001).Genomic fingerprint analysis of DEPArray™ isolated HCC827 cell line spiked in healthy donor blood. Allelic variants for three distinct genomic markers (D18S535, D13S317 and D21S2039) included in *Ampli*1™ STR kit are shown for HCC827 single cell, HCC827 genomic DNA, donor WBC single cell and donor WBC genomic DNA.

The aim of the study was to establish an experimental workflow allowing reliable molecular single cell analysis in a clinical setting within an acceptable time frame. The workflow allows interrupting sample preparation at several steps, first, after cell enrichment and detection and then after whole genome amplification of isolated CTCs (Fig 1A).

To set up the workflow, we processed 79 CTC-positive CellSearch® cartridges of 66 breast cancer patients provided by clinical centers in Munich, Tübingen and Milan. Baseline characteristics of the patients are shown in Table1. After counting, we flushed CellSearch® cartridges and loaded all cells into DEPArray™ cartridges, from which selected cells can be individually sorted into reaction tubes. The overall CTC isolation efficiency is a result of several steps, including extraction from the CellSearch® cartridge, loading onto and detection by the DEPArray™ system as well as single cell recovery. CellSearch® CTC counts ranged from 1 to 892 CTC (mean 73.1, median 22, Supplementary Table S1). CTCs were re-detected by DEPArray™ using the standard CellSearch® criteria to define a CTC event, comprising (i) nearly round or oval morphology with a visible nucleus within cytoplasm; (ii) staining profile (DAPI^+^/Cytokeratin^+^/CD45^−^); and (iii) minimum diameter of 4 μm. To assess the correlation between CellSearch® CTC counts and DEPArray™ CTC re-detection, we had to account for a dead volume of the DEPArray cartridges, as only 9.26 μl out of 14 μl total volume injected were analyzed in the main chamber, that is, about two thirds of the injected volume. In so doing, we found an excellent correlation between the CellSearch® CTC count and the DEPArray™ detection (Spearman's rho correlation, *n* = 79, *r* = 0.94, *P* < 0.00001, Fig 1B and Supplementary Table S1). Computing the mean ratios of observed over expected CTCs (Supplementary Table S1), we determined a mean transfer efficiency of 85% (median 77%, standard deviation ± 49%). Patient CTCs displayed heterogeneous morphology and staining intensity as in previous studies (Allard *et al*, 2004) adding observer variability to the CellSearch–DEPArray™ comparison. Although we lost the CTCs of two samples during the DEPArray™ procedure (as expected from the transfer efficiency), we found more CTC events than originally detected with CellSearch® in three cases. In all these cases, we detected very small events with a diameter of 4 μm as determined by DEPArray™, plausibly explaining why they were not counted in the CellSearch® enumeration.

**Table 1:** Patient characteristics

		CTC enumeration/7.5 ml			
Variable	Subcategory	< 5	< 50	≥ 50	*P*-value*
Clinical stage	Stage I–III (*n* = 8)	4 (50.0%)	4 (50.0%)	0	0.079
	Stage IV (*n* = 56)	13 (23.2%)	22 (39.3%)	21 (37.5%)	
	Unknown (*n* = 2)	1 (50.0%)	0	1 (50.0%)	
HER2 primary tumor	Amplified (*n* = 10)	3 (30.0%)	4 (40.0%)	3 (30.0%)	0.958
	Negative (*n* = 52)	14 (26.9%)	20 (38.5%)	18 (34.6%)	
	Unknown (*n* = 4)	1 (25.0%)	2 (50.0%)	1 (25.0%)	
HR primary tumor	Positive (*n* = 50)	11 (22.0%)	21 (42.0%)	18 (36.0%)	0.272
	Negative (*n* = 14)	6 (42.9%)	5 (35.7%)	3 (21.4%)	
	Unknown (*n* = 2)	1 (50.0%)	0	1 (50.0%)	

* Chi-square test, patients with unknown clinical stage, HER2 or HR status were not included in statistical analysis.

We next assessed the routing efficiency, defined as the success rate of displacing cells from the main chamber of the DEPArray™ chip into the parking chamber, where cells are collected before flushing them into the reaction tube. Median routing efficiency was 94% (range 75–100%, interquartile range 9%) as assessed on a total of 417 cells (246 CTC, 171 WBC) across 20 different experiments comprising a range of 4–48 cells per experiment.

In total, we isolated 510 single CTCs of 64 breast cancer patients and 189 single white blood cells (WBCs, defined as DAPI^+^/Cytokeratin^**−**^/CD45^+^ cells) of 62 patients. The number of isolated single cells per sample ranged from 1 to 68 cells (mean 7.3, median 6) for CTCs and 1–18 cells (mean 2.6, median 2) for WBCs. Additionally, we isolated 39 CTC pools of 21 patients (range 2–20 cells, mean 6.4, median 5) and 77 WBC pools of 61 patients (range 2–73 cells, mean 10.8, median 6). Of all these samples, we amplified the genomic DNA using the *Ampli*1™ WGA kit (Silicon Biosystems SpA), which is based upon genome-wide Mse I digestion (recognizing the sequence TTAA), ligation of a single adaptor with unique sequence and PCR amplification (Klein *et al*, 1999, 2002).

### Single cell recovery by DEPArray™ and sample purity

To fully exploit molecular CTC analysis in a diagnostic workflow, single cells should be isolated free from contaminating white blood cells (WBCs). To assess the efficiency and purity of single cell recovery by the DEPArray™ system, we spiked cells of different tumor cell lines (DLD1, MCF7, HCC-827 or SW480, respectively) into the blood of healthy donors and analyzed the presence and identity of isolated single cells by DNA fingerprinting (*Ampli*1™ STR kit). The use of different genomes enabled us to unambiguously identify contaminating DNA in every cell isolation and DNA preparation experiment. During six independent consecutive DEPArray™ sorting experiments, we isolated 46 single tumor cells and 26 single WBCs and were able to confirm cell presence in 69 of 72 (96%) of samples by detection of STR bands. The failing three single-cell recoveries were completely free of STR alleles. Most importantly, the DNA fingerprint confirmed pure single cell recovery by showing the expected genotype in 100% of recoveries (Fig 1C). Finally, genotyping was also applied to verify the absence of contaminating cells or DNA in analyzing 9 blank (no-cell) recoveries used as internal negative controls.

### Development of a quality control for whole genome amplification

**Figure 2:**
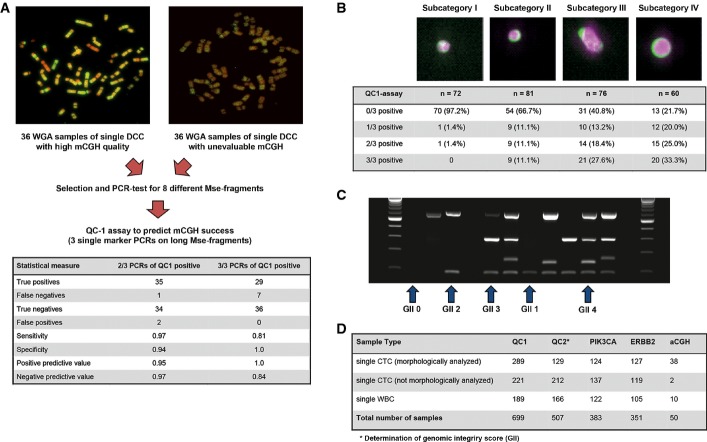
Development of quality control assays for *Ampli*1™ WGA Representative metaphase CGH experiments for successful (left panel) and failing hybridizations (right panel), which were used to identify the three discriminating amplicons. Table summarizes assay performance for the selected primers.CTC subcategories according to size and morphology (examples for subcategory I-IV from left to right) are associated with the detection of the QC1 assay amplicons (*n* = 289, chi-square, *P* < 0.00001).Gel picture of the multiplex PCR assay (QC2 assay). Lanes are loaded (left to right): size marker, MU01 CTC04 (GII 0), MU22 CTC01 (GII 2), MU32 CTC01 (GII 2), TB 04 (GII 0), MU28 CTC04 (GII 3), MU35 CTC04 (GII 4), MU12 CTC05 (GII 1), MU28 CTC03 (GII 3), MU35 CTC01 (GII 2), MU28 CTC02 (GII 4), PCR positive control, PCR negative control, size marker.Summary of sample numbers tested for the different molecular assays.

The quality of molecular data derived from whole genome amplification (WGA) of single cells depends on the integrity of the input DNA. To identify high-quality samples, we devised a test to assess a reliable and comprehensive *Ampli*1™ whole genome amplification (WGA) of single cell DNA. From our existing single cell WGA biobank (with all samples prepared with the *Ampli*1 method), we selected 72 WGA products of single disseminated cancer cells (DCCs) isolated from bone marrow of breast and prostate cancer patients, as well as from lymph nodes of melanoma patients. For each of the three tumor types, we selected 12 DCCs that had been successfully hybridized on human chromosomes in previous metaphase comparative genomic hybridization (mCGH) experiments (*n* = 36) and 36 single DCC WGA libraries that had failed in mCGH experiments (Fig 2A).

We next designed eight primer pairs for Mse I fragments located on different chromosomal regions and with different Mse I fragment length, ranging from 239 to 1936 bp (Supplementary Table S2). All eight specific PCRs were performed on the 72 selected samples, and we found three primer pairs that were associated with successful mCGH experiments. Successful amplification of two out of three or three out of three of these selected Mse I fragments showed a positive predictive value of 0.95 and 1.0, respectively (Fig 2A), for successful karyotyping by mCGH. These three amplicons were then validated using 100 diploid non-cancer cells that had been isolated and their DNA amplified between 1999 and 2008. We selected 22 WGA products of single cells predicted to enable CGH analysis and 10 WGA products of single cells predicted to fail by the selected three primer pairs. The performance of CGH was correctly predicted in all 32 cases, resulting in a first quality control assay (QC1 assay).

We then isolated 88 single mononuclear cells from peripheral blood of a male donor using a micromanipulator, amplified genomic DNA using the *Ampli*1™ WGA kit and assessed the quality of amplification by the QC1 assay. We found that 83 of 88 (94.3%) of the cells showed high DNA quality predictive of a successful metaphase CGH experiment (Supplementary Table S3). For the remaining five cells, we could not detect a single Mse fragment by QC1 indicating loss of the cell during the isolation procedure.

For 22 positive WBC with three positive QC-PCRs, we then tested the allelic discovery rate on few microsatellite and polymorphic loci amenable for restriction length polymorphism analysis (five single nucleotide polymorphisms and five microsatellite repeat polymorphisms). In total, we retrieved 407 of 440 (92.5%) of the 440 expected single copy alleles, summing up to a call rate of 93.6% for microsatellite markers and 91.4% for single nucleotide polymorphisms, respectively (Supplementary Table S4). We concluded that the QC1 assay identifies cells that are suited for comprehensive genome analysis such as CGH and that enable high allelic discovery rates.

### CTC morphology correlates with QC1 assay result

To assess the quality of clinical single cell WGA samples isolated with our workflow, we first determined whether fixation, storage and cell isolation during the CellSearch® and DEPArray™ procedures influence WGA quality. We noted that, while 83 of 88 (94.3%) *Ampli*1™ libraries of freshly isolated, unfixed mononuclear cells of a healthy donor showed high DNA quality, only 124 of 189 (65.6%) WGA libraries of single WBCs isolated from CellSearch®/DEPArray™ cartridges had the same high DNA quality (chi-square, *P* < 0.00001, Supplementary Table S3). Moreover, only 191 of 510 (37.5%) CTCs displayed 2 of 3 or 3 of 3 fragments in QC1 (chi-square, *P* < 0.00001 for comparison with CellSearch-derived WBC, Supplementary Table S3). While the lower DNA quality of fixed versus unfixed WBC most likely reflects fixation or sample processing, the difference between CTC and CellSearch-derived WBCs suggests biological rather than technical underlying reasons.

We re-evaluated the CTC images of the DEPArray™ Cell Browser picture galleries to identify the underlying cause. Since considerable disagreement exists about the correct identification of individual CTC-like events among experienced operators of the CellSearch® system (Kraan *et al*, 2011), we grouped all CTC-like events (defined by the CellSearch criteria mentioned above) into four morphologically distinguishable subcategories. These CTC-like events displayed morphologies from small particles of fragmented cell-like appearance (subcategory I), small events with intact cellular morphology (subcategory II), and large events with irregular cellular morphology (subcategory III) to intensely stained large cells (subcategory IV, Fig 2B). We found CTCs from subcategory I through IV in 23 (55%), 28 (67%), 27 (64%) and 19 (45%), respectively, among 42 analyzed patients. We next correlated the morphological subcategories with the QC1 assay result and observed an increasing WGA quality from CTC subcategory I to subcategory IV (chi-square, *n* = 283, *P* < 0.00001). Importantly, CTCs from subcategory IV displayed similar QC1 results as single WBC recovered from CellSearch® cartridges (Fig 2B, Supplementary Table S3).

### QC2 multiplex PCR assay defines a cell genome integrity index (GII)

Since breast cancer CTCs have been described as being frequently apoptotic (Mehes *et al*, 2001) and since genomic DNA is fragmented into small pieces of 180–200 bp length during Caspase-mediated apoptosis (Wyllie, 1980), we tested *Ampli*1™ WGA products of 252 single CTC and 100 WBC for the presence of a short Mse I fragment of 192 bp length (Supplementary Table S2). Interestingly, this Mse fragment, encompassing the frequently mutated Codon12/13 of the *KRAS* gene, was detected in 23 of 95 (24.2%) isolated single CTC that had been negative for all QC1 assay fragments before, suggesting that these samples contained cellular DNA, which may have been damaged or degraded. Therefore, the non-random nature of our amplification method enables to define a quality control assay consisting of four specific Mse I fragments that assess (i) whether a cell has been successfully isolated (small fragment) and (ii) whether the DNA has been fragmented prior to Mse I digestion (larger QC fragments from the QC1 assay).

With this knowledge, we designed a four marker multiplex PCR assay (QC2 assay), including the three primer pairs of the QC1 assay and primers for the *KRAS* fragment. This multiplex PCR provides a genome integrity index (GII), defined by the detected PCR bands as a measure for quality of each WGA sample generated from an isolated single cell. GII values range from 0 (no band detected) to 1 (only KRAS fragment detected), 2 (any one of the three long Mse fragments detected), 3 (any two of the long Mse fragments detected) and 4 (all three long Mse fragments detected) (Fig 2C).

To validate our multiplex PCR assay, we next compared the results from single marker PCRs of the QC1 with the multiplex results of QC2. In total, 699 WGA samples from single cells had been tested by QC1; of these, we could re-analyze 507 samples by QC2 (Fig 2D). Multiplied by the number of analyzed markers with both assays (*n* = 3; D5S2117, TP53 Ex2/3, CK19), this corresponds to 1,521 evaluable PCR data points. In general, we found a very high concordance between single-plex (QC1) and multiplex (QC2) PCR, confirming 1,472 of 1,521 results (96.8%). However, in rare cases, fragments detected by QC1 could not be detected by QC2 and vice versa.

### Performance of downstream molecular assays

For therapy decisions, knowledge about the mutational state of specific target genes is essential. Furthermore, the karyotype of isolated CTCs may become important. Therefore, we correlated the GII with three different types of molecular single cell analyses to assess its diagnostic utility, including (i) gene-specific point mutation analysis (*PIK3CA* mutations in exon 9 and exon 20), (ii) gene-specific quantification of copy number (*ERBB2* (HER2) amplification) and (iii) genome-wide array CGH (aCGH). The number of single cell WGA samples tested by each assays is given in Fig 2D.

#### Analysis of small sequence changes or point mutations

**Figure 3:**
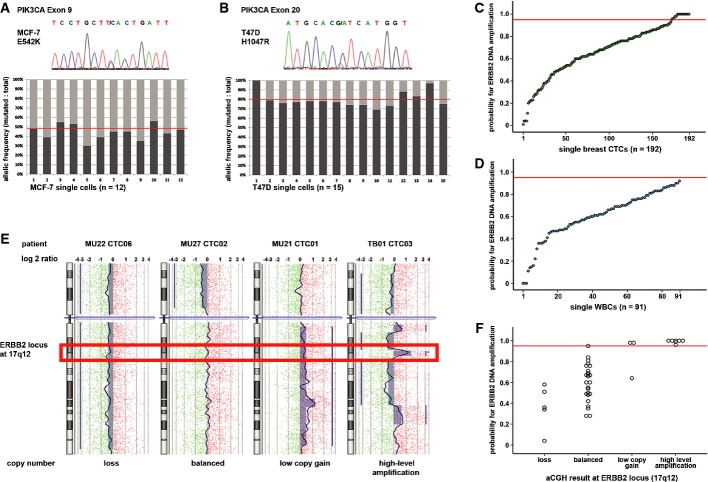
Assays for molecular single cell analysis Exon 9 mutation E545K was detected in all single MCF-7 cells. The mutant allele is representing 45% of detected sequences averaged over all analyzed single cells and 48% in genomic DNA of MCF7 (right). The horizontal red line indicates the allelic ratio of non-amplified genomic DNA.Exon 20 mutation H1047R was detected in all single T47D cells. The mutant allele is representing 80% of detected sequences averaged over all analyzed single cells, as well as in genomic DNA. Cell 01 shows an allelic loss of the wild-type sequence. The horizontal red line indicates the allelic ratio of non-amplified genomic DNA.*ERBB2* copy numbers were assessed by qPCR in 192 CTCs from breast cancer patients. Twenty-one single cells of 7 of 42 patients displayed an amplification probability above 95% (indicated by the red horizontal line).*ERBB2* amplification qPCR determined all single WBCs (*n* = 91) to be negative for *ERBB2* amplification (below the red horizontal line).High-resolution aCGH profiles of four individual cells showing DNA loss (left), balanced aCGH profile (second from left), low copy number gain (second from right) and high-level amplification (right) at *ERBB2* locus (hybridization ratio for single probes shown on a log2 scale).*ERBB2* copy number by aCGH correlates with amplification probability score by qPCR. A qPCR amplification probability score ≥ 0.95 (red horizontal line) indicates *ERBB2* amplification. Two samples dropped out of analysis due to failed amplification of qPCR fragments.

The non-random nature of *Ampli*1™ WGA allows design of specific primers for target sequences of interest based on the distribution of the Mse I restriction-site motif TTAA. *PIK3CA* mutations cluster in two hotspots in exon 9 and 20, which are located on genomic Mse I fragments of 224 and 296 bp length, respectively. After *Ampli*1™ WGA, these mutations are detected in single cells of cancer cell lines with 100% sensitivity and with the expected allelic frequency of 1:1 (wild-type (wt) to mutated (mt)) in single MCF7 and 1:4 (wt:mt) in T47D cells (Fig 3A and B). For clinical samples, targeted Sanger sequencing worked best for high-quality WGAs (GII 3 or 4; chi-square, *n* = 383, *P* < 0.00001, Table2). However, point mutation analysis often also worked with samples displaying a GII of 1 or 2 (up to 89% for *PIK3CA* exon 20; Table2 and Supplementary Table S5). Therefore, gene-specific assay performance clearly depends on the length of the Mse I fragment under investigation.

**Table 2:** Correlation between genome integrity index (GII) and successful performance of different molecular assays

		Genomic integrity index (GII)					
Molecular assay	Analyzed cells	GII 0	GII 1	GII 2	GII 3	GII 4	*P*-value *
PIK3CA Exon 9	*n* = 383	7 of 23 (30.4%)	14 of 25 (56.0%)	48 of 62 (77.4%)	102 of 117 (87.2%)	146 of 156 (93.6%)	< 0.00001
PIK3CA Exon 20	*n* = 383	8 of 23 (34.8%)	18 of 25 (72.0%)	55 of 62 (88.7%)	109 of 117 (93.2%)	149 of 156 (95.5%)	< 0.00001
PIK3CA complete	*n* = 383	4 of 23 (17.4%)	12 of 25 (48.0%)	45 of 62 (72.6%)	97 of 117 (82.9%)	141 of 156 (90.4%)	< 0.00001
HER2 qPCR	*n* = 351	3 of 12 (25.0%)	8 of 18 (50.0%)	41 of 61 (67.2%)	95 of 112 (84.8%)	136 of 148 (91.9%)	< 0.00001
aCGH	*n* = 50	Not assessed	Not assessed	4 of 5 (80.0%)	7 of 9 (77.8%)	36 of 36 (100%)	0.016

* Chi-square test was used to confirm the correlation between performance of molecular assays with genome integrity index. Please note, for raw data on all analyzed single cells see the Supplementary Dataset.

We also addressed the occurrence of sequencing errors. From a previous study, we took sequence data of 46 diploid cells analyzed for 7 loci in *TP53* gene by single-stranded conformational polymorphism method (Klein *et al*, 2002) and had not observed mutations among 128,800 analyzed base pairs. Here, we analyzed 122 single WBCs of 41 patients and 54 WBC pools of 45 patients for mutations in PIK3CA exon 9 and 20 (for success rates in single cells, see Supplementary Table S5). All samples showed wild-type sequence for exon 9 and exon 20, with two exceptions: In one single WBC of patient IB14, we detected a silent mutation (c.3159A > G) near the mutational hotspot of exon 20, and we found a single WBC of patient IB07 harboring a previously not described mutation at codon 1,015 resulting in the exchange of serin to tyrosine. From these data, we estimate a mutation rate of 0.768 in 10^5^ bases, within the range of reported error rates for proof-reading polymerases.

#### Analysis of gene amplifications

In contrast to targeted sequencing, the applied qPCR assay to detect gene amplifications is based on nine different amplicons: three on the target gene, here *ERBB2*, and six on different reference loci. Again, qPCR performed significantly better for GII 3 and 4 samples (chi-square, *n* = 351, *P* < 0.00001; Table2 and Supplementary Table S4). The reproducibility of the assay was shown by high correlation between results of technical replicates (Spearman's rho correlation, *n* = 27, *r* = 0.98, *P* < 0.00001), that is, amplification status of all tested samples could be verified by qPCR repetition (Supplementary Fig S1).

#### Genome-wide analysis of copy number alterations

Finally, we assessed applicability of whole-genome aCGH analysis on single cell *Ampli*1™ WGA samples isolated by our workflow. We first analyzed 10 single CellSearch/DEPArray-derived WBCs of nine patients and noted a higher derivative log2 ratio spread (DLRS) in our single WBC samples (mean 1.28, range 0.64–2.08), as compared to previously published single cell aCGH experiments with unfixed or mildly fixed cells (Czyz *et al*, 2014). The DLRS provides, among other aspects, information about the global signal to noise levels in a sample, with low values indicating better sample and hybridization quality.

We therefore modified our analysis algorithm to address the increase in DLRS. For CellSearch/DEPArray-derived CTCs, we applied analysis settings that ensured that no artificial alterations were called in CellSearch®/DEPArray™-derived WBCs. This was achieved at a resolution of 650 kb, where all analyzed single WBCs displayed a balanced genomic profile (Supplementary Fig S2). Applying these settings to CTCs, we noted that similar to targeted sequencing and qPCR, clinical samples with high GII performed better in whole-genome aCGH (chi-square, *n* = 50, *P* = 0.016, Table2).

To identify the underlying reason for the unexpected increase in DLRS values, we isolated SKBR3 breast cancer cells either unfixed or after CellSearch®/DEPArray™ isolation (for each treatment, three single cells and one cell pool). Isolated cells were amplified by *Ampli*1™ and analyzed by aCGH. We found higher DLRS in CellSearch® treated as compared to unfixed single cells (0.73 and 1.12, respectively), in line with our previous hypothesis that fixation during CellSearch® is influencing genomic integrity and thus performance of *Ampli*1™ WGA. Nevertheless, genomic profiles of untreated and CellSearch® cells applying our algorithm are very similar and correspond to those of genomic DNA, although displaying lower resolution (Supplementary Fig S3).

For cross-assay validation, we compared the measurement for the *ERBB2* locus obtained from qPCR and aCGH. *ERBB2* amplification was detected by qPCR in 21 of 192 single CTC (10.9%) but never in WGA samples of 91 isolated single WBC (Fisher's exact test, *P* = 0.001; Fig 3C and D). We then categorized 47 samples tested by aCGH into *ERBB2* loss, balanced profile, low copy number gain and high-level amplification of *ERBB2* (Fig 3E). *ERBB2* copy numbers by aCGH matched with the qPCR amplification probability score, with only two samples showing discordant results (Kruskal–Wallis test, *n* = 43, *P* = 0.00002, Fig 3F).

#### Genome integrity index and patient stratification

In summary, we found that the GII as determined by QC2 assay correlates with the performance of all molecular assays after single cell isolation and *Ampli*1™ WGA. To determine how many patients are suited for multiple molecular studies, we categorized the patients into two groups. The first group comprises patients with ≥ 5 CTCs in 7.5 ml peripheral blood, which are at increased risk of progression, and the second group patients with < 5 CTCs displaying lower risk of progression. From the first group, we could isolate CTCs with GII 3 or 4 (high-quality DNA) in 68% patients, while high-quality DNA samples could rarely be obtained from patients with low CTC numbers (Fisher's exact test, *n* = 74, *P* = 0.0019). This difference is specific for CTCs, as WBCs (Table3) performed equally well in both groups.

**Table 3:** Detection of high-quality CTCs (GII 3 or 4) in breast cancer patients

Individual CTC with highest GII	GII 0/1	GII 2	GII 3	GII 4	*P*-value*
≥ 5 CTC/cartridge^a^; *n* = 60	14 (23%)	5 (8%)	11 (18%)	30 (50%)	
< 5 CTC/cartridge^a^; *n* = 15	10 (67%)	2 (13%)	0	2 (13%)	
	Low quality		High quality		0.0019
Individual WBC with highest GII	GII 0/1	GII 2	GII 3	GII 4	*P*-value*
≥ 5 CTC/cartridge^a^; *n* = 58	8 (14%)	2 (3%)	10 (17%)	38 (66%)	
< 5 CTC/cartridge^a^; *n* = 14	2 (14%)	2 (14%)	6 (43%)	4 (29%)	
	Low quality		High quality		0.45

a As determined by CellSearch® analysis.

* As determined by Fisher's exact test.

### Heterogeneity in metastatic breast cancer patients

Technical reliability is the *sine qua non* to investigate cancer cell heterogeneity, which may underlie individual treatment responses. Having firmly established the conditions of single cell analysis, we proceeded to interrogate the potential impact of our findings.

**Figure 4:**
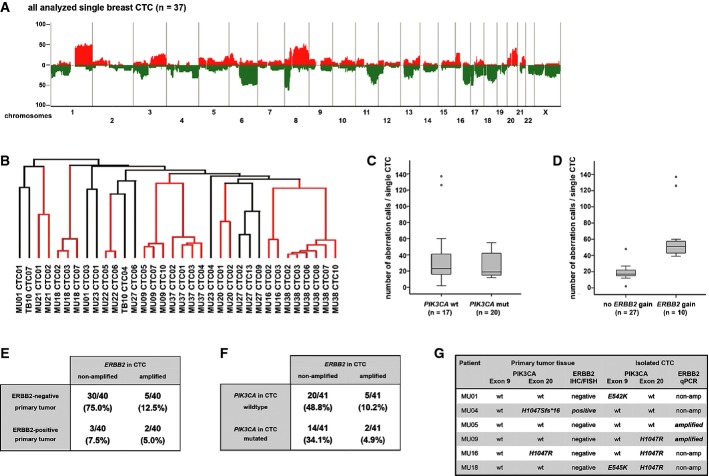
Molecular heterogeneity of breast cancer CTCs A Chromosomal aberrations in 37 single CTCs of 15 breast cancer patients. The histogram plot displays the frequency of genomic gains (red) and losses (green) of CTCs, which are characteristic for breast cancer cells. B Unsupervised hierarchical cluster analysis using the average linkage mode of breast cancer patients with more than one analyzed CTC (34 cells of 12 patients). Distances of vertical lines to the next branching point in dendrogram represent relatedness. Red vertical lines indicate that all analyzed CTCs of an individual patient are located within the same branch of the dendrogram. C, D Number of chromosomal changes in *PIK3CA-*mutated (C) and *ERBB2*-amplified (D) versus wild-type CTCs. Note that CTCs with *ERBB2* amplification showed a significantly higher number of genomic aberrations than CTCs without amplification (*n* = 37, Mann–Whitney *U*-test, *P* < 0.00001). E *ERBB2* status in CTCs versus *ERBB2* status in primary tumors in individual pairs. F *PIK3CA* mutational state and *ERBB2* copy number gains in CTC of individual patients. G Paired analysis of mutational states for *PIK3CA* and *ERBB2* of primary tumors versus matched CTCs of six patients. Note that only patient MU16 displays shared states in primary tumors and CTCs.

**Figure 5:**
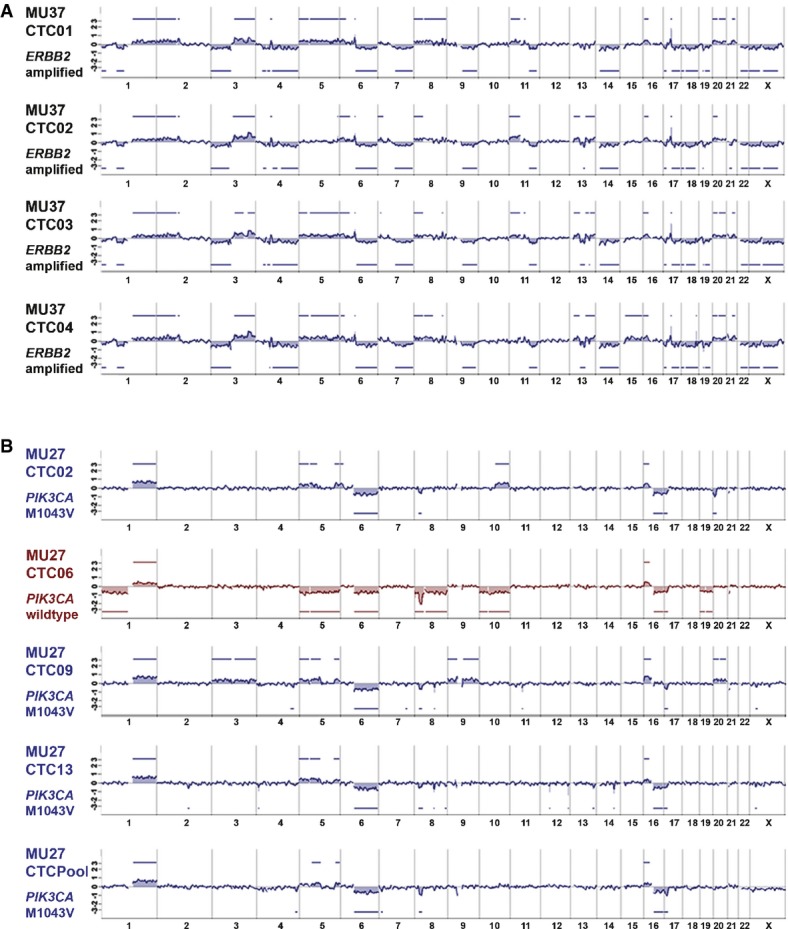
Genomic profiles of single breast cancer CTCs Genomic overview over four isolated single breast CTCs with *ERBB2* amplification (patient MU37). All CTCs of patient MU37 show high number of chromosomal aberrations and high clonality in their genomic profile. *ERBB2* high-level amplification for all CTCs was validated by qPCR assay (aberration calls depicted on *y*-axis in log-2 scale).Genomic overview over four isolated single breast CTCs and a CTC pool from patient MU27. Three of four CTCs show highly similar genomes and M1043V mutations in exon 20 of *PIK3CA* as the analyzed CTC pool (blue profiles). However, one CTC (CTC06, red profile) displays several different chromosomal aberrations (aberration calls depicted on *y*-axis in log-2 scale) and lacks the M1043V mutation.

In a first step, we analyzed 37 single CTC of 15 patients and detected structural chromosomal changes in all analyzed CTC. The detected genomic gains and losses are characteristic for breast cancer (Fig 4A). Cluster analysis revealed patients with varying degrees of clonal similarity. Of note, *ERBB2*-amplified CTCs displayed little genome-wide heterogeneity (Figs4B and 5A). To investigate the impact of specific oncogenic mutations onto the genomic rearrangement of single CTCs, we compared the genomes of cells with *PIK3CA* mutations and *ERBB2* amplifications. We found that *PIK3CA*-mutated CTCs do not differ from *PIK3CA* wild-type CTCs regarding the frequency of copy number alterations (Mann–Whitney *U*-test, *n* = 37, *P* = 0.478, Fig 4C), while *ERBB2*-amplified CTCs displayed significantly higher numbers of genomic aberrations than CTCs without *ERBB2* amplification (Mann–Whitney *U*-test, *n* = 37, *P* < 0.00001, Fig 4D).

This prompted us to have a closer look into the *ERBB2*-amplified and *PIK3CA*-mutated CTCs. In total, 7 of 42 patients (16.7%) displayed *ERBB2* amplification in CTCs. Of these patients, all analyzed single CTCs displayed the *ERBB2* amplification with only one exception indicating rare heterogeneity (14% of patients) among CTCs for this specific aberration (Supplementary Fig S4). However, when we compared the CTC findings with the *ERBB2* status of the primary tumor, we noted that 8 of 40 (20%) patients with available *ERBB2* status of the primary tumor displayed disparity between CTCs and primary site for *ERBB2* amplification (Fig 4E). Moreover, of the 10 patients with *ERBB2* changes in either the primary tumor or the CTCs, eight patients (80%) displayed disparate *ERBB2* amplification states.

We next analyzed the potential therapy target *PIK3CA*. We could assess both *PIK3CA* mutational hot spots in 202 of 261 analyzed CTCs (77.4%, Supplementary Table S5) and at least one of the two exons in 241 of 261 (92.3%). Somatic mutations were detected in 54 of 202 CTCs (26.7%) resulting in 16 of 43 (37.2%) breast cancer patients harboring mutated CTCs. Molecular heterogeneity among *PIK3CA*-mutant CTCs was higher than for *ERBB2*-amplified CTCs and detected in 10 of 16 patients (62.5%; 5Fig and Supplementary Table S6). For seven patients with mutated CTCs, we could additionally amplify CTC cell pools (2–14 cells). Among these samples, the *PIK3CA* mutational status of the CTC pool matched the result of the majority of single cells in six cases (four times mutated, two times wild-type). Of note, in one patient (MU18) who harbored three single CTCs with wild-type *PIK3CA* and one CTC with a H1047R exon 20 mutation, the recovered CTC pool of 14 single cells showed sequences of E545K exon 9 mutation (allelic ratio mt:wt = 30:70; Supplementary Table S6).

Interestingly, CTCs of one individual patient may be heterogeneous for specific mutations, and may be associated with characteristic genomic rearrangements. Of four patients, we analyzed pools of CTCs by aCGH in addition to single CTC samples. Genomic profiles of CTC pools corresponded well with single cell data and confirmed the high clonal relationship between individual CTCs. However, in one patient with 409 CTC in 7.5 ml of blood (MU27), we detected the M1043V mutation in exon 20 of *PIK3CA* in the CTC pool and all but one single CTC. Strikingly, this specific *PIK3CA*-wild-type CTC displayed different genomic aberrations than all other analyzed CTCs (Fig 5B).

Finally, CTCs of 2 of 7 (28.6%) patients with *ERBB2* amplification in CTCs also displayed *PIK3CA* mutations (Fig 4F and Supplementary Fig S4). Assessing the *PIK3CA* mutational status for matched primary tumors and CTCs of six patients, we found a disparity of 66% (Fig 4G). When the combined status of *ERBB2* and *PIK3CA* was assessed for these individual pairs, only 1 of 6 pairs displayed identical patterns in primary tumors and CTCs (Fig 4G).

## Discussion

Here, we provide a workflow for the molecular characterization of CTCs that enables reliable analysis of single cells for diagnostic purposes. We addressed the diagnostic needs of faithful detection of mutations, selected gene amplifications and genome-wide copy number alterations in CTCs by combining the CellSearch® system for CTC enrichment with an automated cell isolation method, the DEPArray™ technology, and the non-random *Ampli*1™ WGA method. Importantly, the developed single cell isolation and molecular analysis workflow is not restricted to CellSearch® enrichment but can easily be applied to other methods which are able to enrich CTCs and provide a cell suspension.

Currently, clinicaltrials.gov lists more than 260 studies, which explore the utility of CTC detection, enumeration and targeted molecular analysis. There is substantial hope that CTC analysis will help to monitor disease progression, predict response to selected drugs and identify therapy-resistant cancer early on. Hence, there is a great medical need to provide a diagnostic workflow that enables clinical decision-making based on few available cancer cells. By all available technologies, the number of CTCs in cancer patients is very low, although some partially conflicting results have been reported. Perhaps, the most reliable data have been provided by the CellSearch® system (Cristofanilli *et al*, 2004) and analysis of diagnostic leukapheresis samples (Fischer *et al*, 2013). The CellSearch® system is the only FDA-cleared CTC detection and enumeration device currently available and has therefore been used in the majority of studies. Its usefulness in breast cancer has been firmly established (Bidard *et al*, 2014), although its dependence on positive selection of EpCAM-positive CTCs may lead to false-negative or false-low CTC results in some patients (Sieuwerts *et al*, 2009). By enrichment-independent analysis of leukapheresis samples, however, it was shown that CTC numbers are generally low ranging from 1 to 15 cells per ml of blood (Fischer *et al*, 2013) at least for breast cancer. This upper level of CTC numbers suggests that standard molecular methods established for thousands to millions of cells need to be adjusted to few or even single cells, if molecular information needs to be obtained.

CTC analysis is likely to become complementary to primary tumor analysis the more disease courses are prolonged by therapy. Molecular diversity among tumor cells has been recognized as a major driving force for evolution of an individual cancer (Greaves & Maley, 2012; Klein, 2013) and takes largely place outside the primary tumour. Today, metastasis frequently arises years after excision of the primary in many breast cancer patients and most metastatic patients experience multiple lines of systemic therapies. The applied iatrogenic selection leads to survival of tumor cells with acquired resistance, one example being *ESR1* mutations under antihormonal therapy (Toy *et al*, 2013). Therefore, the medical need to re-evaluate the molecular profiles of remnant cancer cells is likely to grow with the number of therapeutic options.

### Characteristics of the established workflow

We consequently established and explored a full diagnostic workflow from cell detection to molecular characterization. This workflow can be easily integrated into clinical studies, because it enables several points of discontinuation without loss of information. After CellSearch detection, cartridges can be stored before transfer to the DEPArray for at least 3 weeks. Then, after isolation and WGA, DNA samples can be stored for several years (Czyz *et al*, 2014). More specifically, we found that we can morphologically pre-select CTCs with intact genomes that are best suited for genetic analyses. Large, round CTCs gave rise to excellent molecular results similar to single WBCs, while small and irregularly formed CTCs may represent damaged cells. We uncovered this association because we had developed a simple PCR-based quality control (QC) assay for *Ampli*1™ WGA products that predicted the outcome of downstream molecular studies. The genomic integrity index (GII) deduced from this QC assay classifies all isolated cells into four categories, with cells from categories 3 and 4 being optimally suited for further characterization.

The use of the workflow within clinical studies is further supported by the high information retrieval from rare events. We validated the successful transfer of detected CTCs from the CellSearch® to the DEPArray™ system and isolation into the reaction tube. Transfer efficiency was 85% and isolation efficiency 94%, resulting in a success rate of 80% that a detected CTC can be molecularly explored. Factors determining whether or not the isolated cell will provide high-quality molecular information were tested next. First, cell fixation reduced the percentage of high-quality DNA samples obtained from normal diploid WBCs from 94 to 66% and increased the noise in array CGH experiments. Second, the GII of CTCs was shifted to lower values compared to WBCs with about 38% of CTCs generating high-quality DNA samples. This indicates that a substantial proportion of CTCs are damaged or apoptotic as suggested previously (Mehes *et al*, 2001). Consequently, it was more difficult to generate high-quality DNA samples from patients with < 5 CTCs than from patients with ≥ 5 CTCs. In total, we estimate that samples with ≥ 3 CTCs will enable successful molecular characterization of at least one CTC (0.85 × 0.94 × 0.375). From our patient cohort, we isolated high-quality CTCs from 57% of patients.

The ability to establish the QC assay and the strong correlation between WGA and downstream molecular assays is clearly linked to the deterministic nature of the *Ampli*1™ WGA assay (Klein *et al*, 1999), which has been tested extensively in previous studies using patient samples (Klein *et al*, 2002; Schmidt-Kittler *et al*, 2003; Schardt *et al*, 2005; Weckermann *et al*, 2009; Fischer *et al*, 2013; Ulmer *et al*, 2014). The method has several advantages over single cell amplification methods that employ random priming such as DOP (Telenius *et al*, 1992) or PEP (Zhang *et al*, 1992), MALBAC (Zong *et al*, 2012) or multiple strand displacement (Hou *et al*, 2012). For *Ampli*1™ WGA, the genome is cut at the restriction-site TTAA. Therefore, selected amplicons will be amplified equally in all single cells of all patients, with minor differences among individuals due to polymorphisms in the TTAA motif. CTCs from GII categories 3 and 4 were positive for TTAA-fragments of *PIK3CA* and *KRAS* in more than 90% of cells. CTCs from GII category 1 and 2 may still provide valuable information for mutations in selected genes. For example, more than 50% of cells with GII 2 are suited for analysis of amplicons harboring the hot spot regions of *PIK3CA* exon 9 and exon 20. Likewise, assays for therapy relevant copy number alterations can be easily designed (similar to *ERBB2* locus investigated here) and enable faithful analysis of > 90% CTCs from GII categories 3 and 4. This is in stark contrast to other methods that display allelic discovery rates of about 70% or less (Zong *et al*, 2012). The downside of *Ampli*1™ is that some loci may be less easily accessible if the TTAA-fragments become either too large or too small. However, even this disadvantage can be predicted from DNA sequences of interest and can be overcome easily by selection of a different restriction enzyme for a specific diagnostic question.

Reliable assessment of single CTCs for combined genetic alterations is unique to approaches that analyze individual cells. Unlike other approaches that display low purity of isolated cells (Nagrath *et al*, 2007; Yu *et al*, 2012), we could show by DNA fingerprinting analysis of spike-in experiments that we were able to isolate and amplify genomic DNA of 96% of isolated single cells with 100% purity. Methods that amplify DNA or RNA of CTCs within a background of contaminating normal cells, such as WBCs (Nagrath *et al*, 2007; Yu *et al*, 2012), or other CTCs (Magbanua *et al*, 2012, 2013), or methods that rely on the analysis of circulating nucleic acids (Schwarzenbach *et al*, 2011) will not retrieve cellular combinations of alterations with diagnostic precision.

### Molecular findings in clinical samples

For the first time, we confirmed the malignant origin for all individually isolated breast cancer CTCs. Previous studies investigated pools of cells (Magbanua *et al*, 2012, 2013) or identified cells as aberrant or normal for selected molecular markers (Lowes & Allan, 2014). Since the cell selection for aCGH was only based on the GII, we conclude that CellSearch® criteria-positive and GII-high cells from M1 stage breast cancer patients are indeed CTCs.

In cancer, the unit of selection is the individual cancer cell, and hence, we expect most insight into drug resistance by comparative studies on purified individual CTCs before and after administration of a drug. The potential use of this reasoning is exemplified by our analysis of *ERBB2* and *PIK3CA* alterations. For the *PIK3CA* and *ERBB2* alterations, we determined the reliability of both assays to be > 90% for GII 3 and 4 cells. Because assay-related single cell variation will multiply its negative effects, the more genomic loci of a cell are investigated, such a high reliability of each assay was needed to demonstrate combined alterations of the two genes. In our cohort, we identified 2 out of 7 patients with *ERBB2* amplification-harboring CTCs who had double-mutant *PIK3CA/ERBB2* CTCs prior to administration of *ERBB2*-targeting therapies. This is of interest as somatic mutations in *PIK3CA* were shown to be associated with resistance against *ERBB2*-targeted therapies (Berns *et al*, 2007; Chandarlapaty *et al*, 2012) and significantly worse outcome in *ERBB2*-positive patients (Cizkova *et al*, 2013). Since first studies demonstrate efficacy of PIK3CA pathway inhibitors for these patients (Janku *et al*, 2012), prior assessment of pre-existing resistant cells may become useful. Furthermore, we found that for one individual patient, both *PIK3CA* hot spot mutations may co-exist in different cells, providing further compelling evidence for independent and parallel acquisition of driver mutations by individual cancer cells.

### Insights into the molecular evolution of systemic cancer

Our data show that single cell analysis will enable a better understanding of cancer evolution. Measures of cellular diversity or evolutionary state are a novel form of biomarker, which are increasingly recognized (Maley *et al*, 2006; Park *et al*, 2010) but have only recently been applied to metastasis research (Almendro *et al*, 2014). Evidence is growing that disseminated cancer cells continue to accumulate alterations outside the primary tumor (Schmidt-Kittler *et al*, 2003; Weckermann *et al*, 2009; Klein, 2013) and that the number of genetic alterations in single CTCs is associated with shortened survival (Fischer *et al*, 2013). For our patients, we identified 14% of patients with *ERBB2*-negative primary tumors harboring *ERBB2*-positive CTCs and a disparity in 66% of individual primary tumor–CTC pairs for *PIK3CA* mutations. In only 1 out of 6 patients, CTCs shared the mutational state of the oncogenes *ERBB2* and *PIK3CA* with the primary tumor. As we did not apply subclone analysis on primary tumor samples, we cannot exclude the existence of CTC-associated mutations that are detected in a minority of primary tumor cells, as it has been suggested by recent sequencing studies of primary tumors revealing molecular heterogeneity of different subclones within individual tumors (Gerlinger *et al*, 2012). However, it is noteworthy that we found in most cases *ERBB2* amplifications to be shared homogeneously among analyzed CTCs of individual patients. Consequently, if cells with this specific alteration previously represented a minor subclone within the primary tumor, they had now turned into the predominant clone outside the primary tumor. This suggests that some driver mutations, acquired within or outside the primary tumor, confer selective advantages for survival and expansion of cancer cells *outside* the primary tumor.

We also observed that the degree of genomic rearrangement of a cell is associated with specific driver mutations. *ERBB2*-amplified CTCs displayed higher numbers of genomic alterations than *ERBB2-*negative CTCs, whereas no such effect was seen for *PIK3CA* mutations. Whether the pronounced clonality of *ERBB2*-amplified CTCs from *ERBB2*-negative primary tumors suggests strong oncogene addiction must await further exploration. However, the findings demonstrate that molecular analysis of individual CTCs may uncover evolutionary mechanisms useful for personalized therapy decisions.

Last but not least, CTC characterization with diagnostic precision as described here may provide an answer to the outcome of the SWOG S0500 trial, where CTC enumeration was shown not to be sufficient to inform on alternative chemotherapeutic treatments (Smerage *et al*, 2014). Only molecular CTC analysis linked to targeted therapies may thus represent the means to bring the concept of liquid biopsy to clinical success.

## Materials and Methods

### Patients and CTC enrichment and detection using the CellSearch® assay

Enrichment and detection of CTCs was performed within the SUCCESS (EUDRA-CT number 2005-000490-21) and DETECT (EUDRA-CT number 2010-024238-46) (Fehm *et al*, 2010) studies using the CellSearch® system (Riethdorf *et al*, 2007) and within various studies exploring the role of CTCs at the European Institute of Oncology in Milan between August 2011 and August 2012. Written informed consent for CTC analysis and characterization was obtained for all patients included. All experiments conformed to the principles set out in the WMA Declaration of Helsinki and were approved by the ethical committees responsible for the corresponding studies (Universities of Munich, Dusseldorf, Ulm, and European Institute of Oncology, Milan).

One up to three 7.5-ml blood samples were collected into CellSave® tubes (Veridex Inc.). The CellSearch® Epithelial Cell Test (Veridex Inc.) was applied for CTC enrichment and enumeration according to the instruction from the manufacturer. In brief, CTCs are captured from peripheral blood by anti-epithelial cell adhesion molecule (EpCAM)-antibody-bearing ferrofluid and subsequently checked for positivity or negativity for cytokeratin, the leukocyte common antigen CD45 and 4′,6-diamidino-2-phenylindole (DAPI) staining to ensure the integrity of the nucleus. Samples from the SUCCESS study were prepared using a slightly modified protocol. Here, peripheral blood was drawn into three separate CellSave® tubes (30 ml). The samples were shipped at room temperature to the central cancer immunological laboratory at the University of Munich and analyzed within 96 h of blood sampling. The 30-ml blood samples were centrifuged for 10 min at 800 × *g*. The plasma was removed, and a dilution buffer was added as described. This mixture was overlaid on 6 ml of Histopaque (Sigma, Germany) and centrifuged for 10 min at 400 × *g*. Subsequently, 7.5 ml of this sample containing the buffy coat was processed on the CellTracks® AutoPrep® system using the CellSearch® Epithelial Cell kit (Veridex, USA).

To setup this proof-of-principle study, only CTC-positive cartridges were sent from Munich and Tübingen to Regensburg for cell isolation and mutational analysis (ethics vote number 07-079). Cartridges analyzed by Silicon Biosystems were obtained from the European Institute of Oncology, Milan. Although in clinical studies, only CellSearch® cartridges with at least 5 CTC/7.5 ml of blood are counted as positive, 15 samples with < 5 CTC/7.5 ml were included into this study to better evaluate the workflow.

### Isolation of CTCs and WBC by DEPArray

Cells were extracted from CellSearch® cartridge using a 200 μl gel-tip pre-rinsed in PBS-BSA 2% and transferred to a new protein LoBind 1.5-ml tube (Eppendorf, Germany). Subsequently, the cartridge was washed two times using 325 μl of SB115 Buffer (Silicon Biosystems SpA, Italy) and repeatedly pipetting against the inner surface. The complete fluid was transferred to the 1.5-ml sample tube. After centrifugation at 1,000 *g* for 5 min in a swinging-bucket rotor centrifuge, the supernatant was discarded, 1 ml of SB115 Buffer was added, and the tube was again centrifuged at 1,000 *g* for 5 min in a swinging-bucket rotor centrifuge. The supernatant was withdrawn, and the pellet was resuspended in a final volume of 14 μl SB115 Buffer.

Next, the sample was loaded on a DEPArray™ chip and scanned for CTC to isolate cells for molecular analysis according to the manufacturer's protocol. Cell suspensions from ten cartridges of individual patients with high CTC counts were split into two or more aliquots in order to increase the number of recoveries. This was initially necessary as the number of single cell recoveries was limited to 16–18, while the current version of the DEPArray™ allows up to 35 recoveries per run. Recovery of a single cell by DEPArray™ system takes 8–10 min. The current version of the system needs about 3 min for a single cell recovery.

In order to reduce the collected volume of 18 μl SB115 Buffer to 1 μl of PBS required for *Ampli*1™ WGA, tubes containing recovered single cells were centrifuged at 14,100 *g* for 30 s in a fixed rotor centrifuge. 100 μl of 1× PBS was added without disturbing the sample, and tubes were centrifuged at 14,100 *g* for 10 min in a fixed rotor centrifuge. To reach the final volume of 1–2 μl containing the isolated cell, a 200-μl pipette with its tip pointing to the tube wall opposite to the centrifugation direction was used. The buffer was aspirated carefully while sliding the tip on the tube wall and following the air-liquid meniscus toward the tube bottom without dipping the tip.

### *Ampli*1™ whole genome amplification

DNA of isolated cells was amplified using the *Ampli*1™ kit (Silicon Biosystems) according to the recommendations of the manufacturer resulting in 50 μl of WGA product. The method is based on a published adaptor-ligation-mediated whole genome amplification protocol (Klein *et al*, 1999, 2002).

### DNA fingerprinting of *Ampli*1™ WGA samples

DNA fingerprinting using short tandem repeat (STR) was carried out using an STR-based assay, encompassing a multiplex PCR with 11 loci compatible with *Ampli*1™ WGA digest according to the manufacturer's manual (*Ampli*1™ STR kit, Silicon Biosystems, Spa). STR analysis was used to confirm purity of isolated single cells *Ampli*1™ WGA DNA libraries in spiking experiments and additionally used to exclude exogenous DNA in blank controls of patient samples.

### *Ampli*1™ WGA quality control assays (QC1 and QC2 assay)

Single marker PCRs for detection of 8 specific Mse fragments for the development of QC1 assay were conducted as previously described (Schardt *et al*, 2005). For all PCRs, 0.5 μl WGA product was used as template; oligonucleotide sequences and corresponding annealing temperature T_A_ are listed in Supplementary Table S2. Single cell metaphase CGH was done according to Klein *et al* (2002, 1999) to validate the selected primer pairs for the QC1 assay.

For the multiplex PCR (QC2 assay), 1 μl WGA template was used in 10 μl of a water-based mastermix containing 1× FastStart PCR Buffer (including MgCl_2_), 200 nM dNTPs, 0.5 U FastStart Taq Polymerase and 4 μg BSA (all consumables Roche Diagnostics GmbH, Germany). The eight primers of QC2 assay (KRAS, D5S2117, TP 53 Exon 2/3 and CK19, see Supplementary Table S2) were each used in an end concentration of 0.4 μM. PCR was started with a first step at 95°C for 4 min, followed by 32 cycles of 95°C for 30 s, 58°C for 30 s and 72°C for 90 s, and a final elongation step of 7 min at 72°C. To determine the genome integrity index, PCR products were visualized on a 1.5% agarose gel. The protocol of the multiplex PCR assay is the basis for the now commercially available *Ampli*1™ QC kit (Silicon Biosystems spa).

### *ERBB2* qPCR assay and *PIK3CA* sequencing of single cells

To assess *ERBB2* copy number changes, we modified the assay described by Schardt *et al* (2005). Briefly, we assessed the abundance of three Mse fragments from the genomic location of the target gene of interest (here *ERBB2*) by quantitative PCR (qPCR). Additionally, qPCRs were performed with additional primer pairs amplifying six reference genes which are located on different chromosomes and in regions with relatively rare copy number changes in single DCCs of various cancers. Quantitative PCR (qPCR) was performed using a LightCycler 480 (Roche, Mannheim, Germany) and Fast Start Master SYBR Green I Kits (Roche) using 1 μL of primary PCR products from the whole genome amplification diluted 1:100 in H_2_O. The reaction was performed in LightCycler 480 Multiwell Plates 96 (Roche), with a primer concentration of 0.4 mM. Relative quantification analysis was done using the LightCycler 480 software release 1.5.0 (Roche). qPCR was carried out as follows: pre-incubation at 95°C for 5 min, followed by 38 cycles of amplification at 95°C for 20 s, annealing for 15 s at the specified temperature of the corresponding primer pair (Supplementary Table S7) and 15 s at 72°C. Melting curve analysis was carried out between 50 and 95°C with five acquisitions per °C. A previously established external standard curve was used to calculate the PCR efficiency for each primer pair. Measurements showing unspecific products in the melting curve analysis were discarded from further statistical analysis. All samples were run in duplicates and only included into the additive model if they had at least two of three successful measurements of the target and four of six successful measurements of the references.

The LightCycler software provided calibrated ratios of target and reference qPCR values according to Pfaffl (2001). For all qPCR reactions, the same calibrator, consisting of pooled single cells, was used. Using three targets and six references resulted in 18 pairwise ratios per sample that were subsequently transformed to the log2 scale. From these values, qPCR summary statistics were computed using the “median polish” method. The obtained result was statistically compared to the normal distribution of values obtained by the measurement of 130 WGA-amplified diploid single cells. Amplification probability was defined as one minus the type I error for classifying a normal control cell as being amplified. The resulting score ranges from 0 to 1, and an amplification probability of 0.95 or higher defined the tested cell as amplified for *ERBB2*. All statistical analyses were performed using the R programming language (R-Development-Core-Team, 2011).

*PIK3CA* mutation was assessed by *Ampli*1™ PIK3CA Seq kit (Silicon Biosystems spa) on single cells following *Ampli*1™ WGA. For each *PIK3CA* exon, 1 μl of WGA product was used for the PCR. Resulting PCR products were loaded on a 1.5% agarose gel, and positive samples were purified using QIAquick purification kit (Qiagen, Germany) according to the manufacturer's protocol with the exception that eluation at the end of the protocol was in 25.0 μl water. Purified samples and primers were then sent to a sequencing provider (Sequiserve, Vaterstetten, Germany). Negative PCR results were considered dropouts for *PIK3CA* analysis.

### Single cell aCGH analysis

For whole-genome analysis, we applied a recently established protocol for aCGH of *Ampli*1™ products from single cells (Czyz *et al*, 2014). Briefly, *Ampli*1™ WGA product is re-amplified three times independently (each 1 μl of *Ampli*1™ product in 50 μl reaction volume), and re-amplification products are pooled after quality control by *Ampli*1™ QC kit. Then, each WGA product is labeled with fluorescence dye-conjugated dCTP and dUTP by two independent PCRs (Cy3 for test and Cy5 for reference sample). As a reference, we used four re-amplified high-quality WGA products of single diploid lymphocytes of a female healthy donor that were pooled. The two labeling reactions (test and reference) are pooled and purified using Amicon Ultra 0.5 columns. DNA yields and dye incorporation rates were quantified using the NanoDrop ND-1000.

Array CGH was performed on oligonucleotide-based SurePrint G3 Human CGH 4 × 180K microarray slides (design code: 022060) according the protocol provided by the manufacturer (Agilent Oligonucleotide Array-Based CGH for Genomic DNA Analysis, version 7.1, December 2011) with slight modifications (Czyz *et al*, 2014). Finally, slides were scanned using an Agilent Microarray Scanner Type C, and images were processed with Agilent Genomic Feature Extraction Software (version 10.7) and imported and analyzed with Agilent Genomic Workbench Software (version 6.5 lite). For defining aberrant regions, we used the ADM-2 algorithm with threshold set to 7.0 and a centralization of 6.0. To avoid false-positive calls, the minimal number of probes in an aberrant interval was set to 50 probes and minimum log2 ratio to 0.25. Considering the average spacing of probes on the microarrays (13 kb), the mean size of detectable genomic aberrations is therefore estimated to be 650 kb.

All data have been deposited in NCBIs Gene Expression Omnibus (GEO, http://www.ncbi.nlm.nih.gov/geo/) and assigned series accession number GSE58192.

### Data evaluation

All statistics were calculated using IBM SPSS Statistics 20 for Windows. Statistical significance was assumed for *P* < 0.05, with all tests performed two-sided. For aCGH evaluation, we used the ADM-2 algorithm of the Agilent Genomic Workbench software.
