# Supplementary material for: Molecular profiling of single circulating tumor cells with diagnostic intention
Source: EMBO Mol Med. 2014 Oct 30;6(11):1371–86. doi: 10.15252/emmm.201404033 (PMC4237466; doi:10.15252/emmm.201404033)
Supplement: Supplementary file 1 [file emmm0006-1371-sd1.pdf]

EMBO
Molecular Medicine

Molecular profiling of single circulating tumor cells with diagnostic intention

Bernhard Polzer, Gianni Medoro, Sophie Pasch, Francesca Fontana, Laura Zorzino, Aurelia Pestka,Ulrich Andergassen, Franziska Meier-Stiegen, Zbigniew T Czyz, Barbara Alberter, Steffi Treitschke,Thomas Schamberger, Maximilian Sergio, Giulia Bregola, Anna Doffini, Stefano Gianni, Alex Calanca,Giulio Signorini, Chiara Bolognesi, Arndt Hartmann, Peter A Fasching, Maria T Sandri, Brigitte Rack, Tanja Fehm, Giuseppe Giorgini, Nicolò Manaresi, and Christoph A Klein

Corresponding author: Christoph Klein, Experimental Medicine and Therapy Research - University of Regensburg

Review timeline:

Submission date:	05 March 2014
Editorial Decision:	24 April 2014
Revision received:	08 August 2014
Editorial Decision:	19 September 2014
Revision received:	25 September 2014
Accepted:	29 September 2014

Transaction Report:

(Note: With the exception of the correction of typographical or spelling errors that could be a source of ambiguity, letters and reports are not edited. The original formatting of letters and referee reports may not be reflected in this compilation.)

Editor: Roberto Buccione

1st Editorial Decision 24 April 2014

Thank you for the submission of your manuscript to EMBO Molecular Medicine. We have now heard back from the three Reviewers whom we asked to evaluate your manuscript. In this case we experienced unusual difficulties in securing three willing and appropriate reviewers. Further to this, one Reviewer delivered his/her evaluation with considerable delay, notwithstanding our efforts to accelerate the process. For this we are very sorry.

You will see that all three Reviewers, with varying degrees, express very significant concerns that prevent us from considering publication at this time.

Reviewer 1, while appreciating the overall potential, points to relevant issues that question actual feasibility in a clinical setting and suggests that the approach might be applicable to only a very limited subset of patients. The Reviewer asks crucial questions on the workflow, as for example the turnaround time and how many CTCs with good genomic integrity need to be actually captured. Finally, Reviewer 1 also questions a technical issue related to the significance of the erbB2 qPCR analysis.

Reviewer 2 lists several important items of concern, both technical and regarding presentation of the results, which all require your action and are important to establish the clinical validity of your

work. Most importantly, and similarly to Reviewer 1, s/he also maintains that overall clinical relevance/feasibility (which after all is the whole point of the manuscript) is not proven.

Reviewer 3 is globally more positive but, again, does raise an important technical issue, namely s/he challenges the actual usefulness and validity of the proposed genomic integrity index. This Reviewer also raises a few other points that require your action.

As mentioned above, the issues raised are of a fundamental nature (with many shared by the Reviewers) and must be fully addressed to the satisfaction of the Reviewers. I would also like to point out that it is of the essence, beyond the fundamental technical/methodological issues, which clearly also need to be solved, that you provide a definite and solid case for the clinical feasibility and relevance of your approach. Without the latter aspect, your manuscript cannot be considered further.

Considered all the above, while publication of the paper cannot be considered at this stage, we would be prepared to consider a suitably revised submission, with the understanding that the Reviewers' concerns must be fully addressed as outlined above with additional experimental data where appropriate and that acceptance of the manuscript will entail a second round of review.

Please note that it is EMBO Molecular Medicine policy to allow a single round of revision only and that, therefore, acceptance or rejection of the manuscript will depend on the completeness of your responses included in the next, final version of the manuscript.

Since the required revision in this case appears to require a significant amount of time, additional work and experimentation and might be technically challenging, I would therefore understand if you chose to rather seek rapid publication elsewhere at this stage. Should you do so, we would welcome a message to this effect.

As you know, EMBO Molecular Medicine has a "scooping protection" policy, whereby similar findings that are published by others during review or revision are not a criterion for rejection. However, I do ask you to get in touch with us after three months if you have not completed your revision, to update us on the status. Please also contact us as soon as possible if similar work is published elsewhere.

I look forward to seeing a revised form of your manuscript in due time.

***** Reviewer's comments *****

Referee #1 (Comments on Novelty/Model System):

See comments

Referee #1 (Remarks):

The authors report on the molecular characterization of CTCs for ERBB2 amplification, PIK3CA hotspot mutations and also report aCGH results on a subset of CTCs.
Overall, it is a nice work that shows the ability of this approach to detect multiple genomic aberrations (e.g. HER2 amplification and PI3K mutations) on single cells.
However, I have several concerns highlighted below:
1. The authors do not mention how many patients they have screened in order to have 66 patients with detectable CTCs. This is crucial for the applicability of their approach. Knowing that only half of metastatic breast cancer patients have at least 5 CTCs/7.5ml of blood and only 38% have at least 10 CTCs/ 7.5ml by CellSearch (Nejm Cristofanilli et al) and knowing that a 40% cell loss is to be expected during the Deparray procedure (Peeters et al BJC 2013), then only a subset of breast cancer patients can be analyzed using this workflow. Now, if we also consider the data provided by Polzer et al in the present paper then the subset of patients will be significantly lower. Indeed, only 37.5% of 510 CTCs had a very good genomic integrity index for downstream molecular analysis corresponding to 79% of their patient population. Thus, it is obvious that the workflow suggested by

Polzer et all can be applicable to only a small subset of metastatic breast cancer patients raising concerns for the applicability of this approach as a tool for clinical testing.
2. Concerning the qPCR assay for erbb2 amplification, the authors need to explain how they have defined the cut-off for positive vs negative results. If they have defined the cut-off based on these series of 192 CTCs/ 91 WBCs then, they need to validate the same cut-off in an independent series of CTCs and WBCs to reveal its true performance.
3. The discrepancy between primary tumors and CTCs is based on a few patients only 6 for the PI3K mutations. Moreover, as the authors acknowledge, this discrepancy could be due to the fact that they have not applied deep sequencing and subclonal analysis of primary tumors.
4. Moreover, there are several questions that arise based on their results that the authors could have discussed. If this workflow is to be tested in a clinical trial, what is the turnaround time to have the results? How many CTCs with very good genomic integrity index one needs to analyze to capture the dominant tumor clone? Just one or two CTCs are enough? Or one needs to consider only cases where molecular analyses for CTCs are available for at least 5 CTCs? What is the meaning of one mutated CTC among 10 wild type CTCs?

Referee #2 (Comments on Novelty/Model System):

Please see remarks to the author

Referee #2 (Remarks):

Major comments
- The authors make the assumption that single cell analysis is more desirable than analysis of populations of cells, and they describe their strategy as "with diagnostic intention." However, this assumption is highly debatable, especially vs. pure populations of cancer cells. Although single cell analysis may begin to reflect heterogeneity, analysis of a population provides greater statistical power and less bias. Furthermore, current tumor diagnostics, ranging from pathology/H&E, IHC, FISH, expression arrays, mutation testing etc. do not use single cell analysis for multiple reasons beyond technical limitations alone. It is in fact completely standard to evaluate tumors based on the observable phenotypes present, and to make therapeutic decisions accordingly. Thus, theoretical pros and cons of single cell assays should be discussed.
- The authors' own data seems equivocal as to the utility of single cell analysis. They should more clearly indicate where single cell analysis provided more useful, clinically relevant biomarker information in this study (i.e, "with diagnostic intention").
- The performance characteristics of each assay tested were not well discussed. This is highly problematic since this report is primarily a feasibility study. For example, the abstract states a high number of single cells subjected to WGA, however, not all were successfully profiled. What was the success rate for each of the assay tested (i.e., sequencing, qPCR, and CGH)? What were the control quality measures for each of the assay? Please provide a section discussing performance characteristics of each assay tested (i.e., sequencing, qPCR, and CGH). Please provide a flow chart for each assay to illustrate how many sequencing, qPCR, and CGH data were excluded and how many were evaluable for the analysis.
- The dynamic range of the log2 ratio values in CGH samples is large (Figure 2C); hence, the quality of the CGH data is in question. Also, despite the low signal-to-noise ratio, the authors were able to assess copy number calls. They should discuss clearly how the microarray data was processed and analyzed without over-fitting the data.

Minor comments
Abstract
- Please state how many of the 510 single CTCs and 189 leukocytes WGA-amplified DNA samples were evaluable/informative for each of the assay tested (sequencing, qPCR, and/or CGH)? How many of the 66 patients had evaluable CTCs?
- Please clarify the statement "We defined a GII...in more than 90% of single cells". Is the claim true all for 510 single CTCs or for a smaller subset of cells belonging to GII category 3 and 4? This claim should be qualified to accurately reflect success rates.

Results
- (Paragraph 2, second sentence) What was the % yield (mean, median +/- s.d) after transferring enriched samples from CellSearch to DEParray cartridges?
- (Paragraph 3) Figure 1C. The example provided is from a cell line. Please provide example of genotyping results from patients' CTCs.
- (Paragraph 4) Please provide a supplementary table showing the breakdown of the 510 CTCs and 189 leukocytes WGA-amplified samples to each of the 66 patients. Were all these samples subjected to WGA QC control? What were the genome integrity indexes (GII) for each cell? Which subcategory (I-IV) does each cell belong? Please indicate what downstream molecular assay(s) was performed for each cell (sequencing, qPCR, and/or CGH). Also please indicate the HER2 status of each patient for comparison with qPCR results.
- (Paragraph 6) Please provide evidence for the statement "...WGA products comprehensively covered the single cell genome."
- (Paragraph 10). The relationship between the numbers mentioned in the text and those found in Figure 1D is not clear. Please clarify connection between text in the manuscript and Figure 1D.
- (Paragraph 10) Does GII of 3 and 4 correspond to 2/3 and 3/3? Please provide consistency regarding GII nomenclature throughout the manuscript including tables and figures as well as supplementary materials.
- (Paragraph 11). Figure 2A and 2D. The threshold for ERBB2 amplification probability score needs to be calibrated using breast cell lines with known HER2 copy number (such as those reported by your group in Czyz, PLOS ONE 2014), including MCF7 (non-amplified HER2).
- (Paragraph 11). Figure 2C. Please clarify if the scale is log ratio or log2 ratio.
- (Paragraph 11). Figure 2E. What is the total of patients? The text mentions 42 while the table shows 40? Please clarify.
- (Paragraph 11). Figure S2. What do the gray boxes represent?
- (Paragraph 12). Please elaborate on the method used for PIK3CA sequencing (Ampli1 PIK3CA Seq Kit). How were the sequencing results validated?
- (Paragraph 13) Figure 4A. Please provide in the Discussion section a comparison of your CGH results with previous CGH data on breast CTCs (e.g. Magbanua et al 2013).
- (Paragraph 13) Figures S3 and S4. For consistency with Figure 2C, please superimpose the distribution of log2 ratios of probes (in genomic order) with the copy number calls (smoothed data). Please label Y-axis (log2 ratio scale).
- (Paragraph 13) What does the phrase "genome shaping" mean? Please clarify.

Additional comments
- What are the concordance rates of replicates obtained from the same amplified DNA material? Please provide data on reproducibility of technical replicates for each of the assays: sequencing, qPCR, and CGH? Please provide correlation analysis of CGH log2 ratio data between single cells and pools of cells (if available) from the same patient.
- How does the Ampli1 WGA method compare with other WGA protocols, e.g. WGA4 (Sigma), adapter-linker-PCR, DOP-PCR and Phi29 rolling-circle-amplification? Please provide data or discussion.

Discussion
- (Paragraph 3) It is unclear what the phrases "deterministic nature" of the Ampli1 WGA assay and "this disadvantage is deterministic" mean. Please explain.
- (Paragraph 3) Please correct TTAA motive to TTAA motif.
- (Paragraph 4) The phrase "The diagnostic relevance of assay-related single cell variation is multiplied..." is a premature assessment since the clinical relevance of single cell diagnostics is currently unclear. Please rephrase.

Methods
- Please provide a time frame for patient enrollment. How many patients were assessed, how many were evaluable?
- Please explain in detail how the enriched suspension from CellSearch is counted, and then transferred and loaded to the DEParray system. What is the original volume from CellSearch? How was the volume reduced to 14uL? Please specify centrifugation parameters if performed. Please list important considerations undertaken to reduce cell loss. Also, the method cited as reference 4 in the Expanded View Results, appears to be a conference abstract only. Since this methodology is central to the present manuscript, the authors should provide a full publication and/or a detailed description in this manuscript.
- Please provide the throughput of single cell acquisition. How many single cells can be captured in given amount of time?

- A published report from this group (cited as Czyz et al accepted for publication in PLOS One) was mentioned in the Expanded View section. The methods in your current work seemed to be heavily based on this report. Please discuss and cite in the main manuscript and provide an updated citation.
- Please provide the PCR primer sequences for the GII multiplex assay.
- Please deposit MIAME-compliant CGH microarray data and associated clinical data/annotation to GEO or other publicly available database. The CGH data smoothing algorithm must also be made available to other researchers.

Referee #3 (Remarks):

Molecular profiling of single circulating tumor cells with diagnostic intention
Polzer et al.

Overall comments

In the Polzer et al paper the authors state they set to overcome 2 obstacles:
1) The isolation of individual tumor cells to purity without contaminating white blood cells (WBCs)
2) Comprehensive analysis of single cell genomes or phenotypes for diagnostic purposes

As such they provide a very useful extensive analysis and summary of breast cancer CTC evaluation which deserves publication pending some amendment and editing.

A major part of the approach is a quality control assay which they call the genome integrity index (GII) based on the detection of four specific amplified Mse I fragments. From the GII they generate a value of 0-4 which provides a measure for quality of each WGA sample generated from an isolated single cell. Although useful this is at best a guide since only 4 loci is a bit crude and subject to noise, limitations that need to be acknowledged.

They also state that they can preselect CTCs by morphology but it needs to be made clearer in the main body of the paper how morphological selection is achieved.

Specific comments:

Page 6
The authors state... "only 124/189 (65.6%) WGA libraries of single WBCs isolated from CellSearch(r)/DEPArrayTM cartridges had the same high DNA quality (chi-square P < 0.0001, Table S3). Moreover, only 191/510 (37.5%) CTCs displayed 2/3 or 3/3 QC-assay fragments (chi-square, P < 0.0001 for comparision with CellSearch-derived WBC, Table S3)."

Comment: The authors need to more clearly define what criteria they are using for genome integrity.

Page 7
The authors state..."We found CTCs from subcategory I through IV in 23 (55%), 28 (67%), 27 (64%), 19 (45%), respectively, among 42 analyzed patients. Since 33 (79%) patients harbored cells of category III or IV, which mostly display a GII of 3 or 4, it will be possible to generate high quality WGA from the majority of patients in clinical studies. "

Comment: Not clear - I could not understand what was said - needs clarifying.

Table 2: Correlation of genome integrity index (GII) and successful performance of different molecular assays"

Comment: Not clear what the p values refer to.

1st Revision - authors' response 08 August 2014

Referee #1 (Remarks):

The authors report on the molecular characterization of CTCs for ERBB2 amplification, PIK3CA hotspot mutations and also report aCGH results on a subset of CTCs. Overall, it is a nice work that shows the ability of this approach to detect multiple genomic aberrations (e.g. HER2 amplification and PI3K mutations) on single cells.
However, I have several concerns highlighted below:

1. *The authors do not mention how many patients they have screened in order to have 66 patients with detectable CTCs. This is crucial for the applicability of their approach. Knowing that only half of metastatic breast cancer patients have at least 5 CTCs/7.5ml of blood and only 38% have at least 10 CTCs/ 7.5ml by CellSearch (Nejm Cristofanilli et al) and knowing that a 40% cell loss is to be expected during the Deparray procedure (Peeters et al BJC 2013), then only a subset of breast cancer patients can be analyzed using this workflow. Now, if we also consider the data provided by Polzer et al in the present paper then the subset of patients will be significantly lower. Indeed, only 37.5% of 510 CTCs had a very good genomic integrity index for downstream molecular analysis corresponding to 79% of their patient population. Thus, it is obvious that the workflow suggested by Polzer et al can be applicable to only a small subset of metastatic breast cancer patients raising concerns for the applicability of this approach as a tool for clinical testing.*

Reply: CellSearch® is currently the only FDA-approved blood test for CTC detection and used in over 100 studies that evaluate CTCs as biomarker (information provided upon request by Janssen Diagnostics). These facts and the availability of the system in clinical centers were the rationale behind choosing CellSearch® for CTC enrichment and detection. As the reviewer correctly states, CellSearch® has a limited detection rate in only half of metastatic breast cancer patients at a cut-off level of at least 5 CTC/7.5 ml of blood. Applying this cut-off, CellSearch® at the moment is by far the best validated method of single CTC detection in breast cancer with good correlation with prognosis in large cohorts of patients even for small numbers of CTC (Bidard et al. Lancet Oncology 2014). Validation of the CellSearch® technology was not part of our study. Thus, only CellSearch® cartridges positive for CTCs (as evaluated by the experienced clinical centres) were shipped to the experimental centres in Regensburg and Bologna. We would however, like to emphasize that our downstream analysis is not depending on the CellSearch® system, but only on the ability to detect, isolate and analyze a single cell.

On the other hand, the natural limitation of the approach is that CTC numbers in most patients are low. The probably most reliable study to assess the number of CTCs per patient is the unbiased approach (depending only on cytokeratin as marker) using leukapheresis samples (Fischer et al. PNAS 2013). Here it was found that while up to 90% of breast cancer patients may become CTC positive after hematopoietic stem cell (and possibly cancer cell) mobilization, the total (!) medium number of CTCs per patient is around 7500, i.e. little more than 1 CTC per ml. However, even a rare biomarker may be informative and highly relevant. Therefore, to address feasibility of the workflow to samples with low CTC count, we additionally included 15 cartridges with less than 5 CTC/7.5 ml into the study.

To make the important validation experiments of the workflow more visible in the manuscript and clarify the recruitment strategy, we rephrased multiple paragraphs in the main text, moved results from the supplementary to the main body of the manuscript. Additionally, we present the experimental concept of the study in a new flowchart (new Figure 1A),and included data on the number of isolated CTCs for each cartridge in Table E1. Finally, we provide the overall success rate and estimate that our approach will provide valuable molecular information of at least one CTC in patients with ≥3 cells.

2. *Concerning the qPCR assay for erbb2 amplification, the authors need to explain how they have defined the cut-off for positive vs negative results. If they have defined the cut-off based on these series of 192 CTCs/ 91 WBCs then, they need to validate the same cut-off in an independent series of CTCs and WBCs to reveal its true performance.*

Reply: The cut-off value of the qPCR assay was defined in a single cell study on cancer cell line cells with known ERBB2 FISH-state and disseminated cancer cells of esophageal cancer patients that is part of a separate study currently finalized in a manuscript. As we agree that this information is needed to better follow and judge the performed qPCR experiments for this study, we have summarized the relevant results of this second study and attached a confidential file.

We furthermore added information in the methods section and Table E7 comprising the primer sequences. Additionally, we performed an experiment analyzing the *ERBB2* amplification probability in technical replicates of single cell WGA samples (Figure E1).

3. *The discrepancy between primary tumors and CTCs is based on a few patients only 6 for the PI3K mutations. Moreover, as the authors acknowledge, this discrepancy could be due to the fact that they have not applied deep sequencing and subclonal analysis of primary tumors.*

Reply: We agree with the reviewer that the discrepancy between primary tumour and CTCs is very interesting; however, investigating its cause is beyond the scope of the manuscript. First of all, currently the clinical impact of low abundant mutations within the primary tumour tissue remains unclear. Secondly, molecular diversity between tumour cells has been recognized as a major driving force for evolution of an individual cancer (Greaves and Maley, Nature 2012). Today, metastasis frequently arises years after excision of the primary in many breast cancer patients and the individual cell/subclone harboring a certain mutation of interest remains unknown. Moreover, most metastatic patients experience multiple lines of systemic therapies putting selective pressure on surviving tumor cell clones constantly shaping their genomic repertoire and recent data on ESR1 mutations under therapy selection strengthen the clinical impact of these evolutionary forces (Toy et al. Nature Genetics 2013).

These considerations suggest that the primary tumour will become increasingly less relevant the longer disease courses last and the more iatrogenic selection pressures are exerted. Deep sequencing of the ENTIRE primary tumour is NOT clinical practice and will at least in Germany very unlikely be part of the clinical routine due to laws requiring storage of paraffin-blocks for at least 10 years. Our sequencing approach has the power to detect mutations present in about 10% of the cells within the analyzed sample and therefore would also capture rare events. Finally, we would like to mention that we analyzed breast cancer and not renal cancer in contrast to the Gerlinger study (Gerlinger, NEJM 2012). The average diameter of breast cancer at diagnosis in Germany is currently about 1.3 cm, hence much smaller than the sample in the Gerlinger NEJM study. Thus, isolating DNA from the sections should capture most of the prevalent mutations.

4. *Moreover, there are several questions that arise based on their results that the authors could have discussed. If this workflow is to be tested in a clinical trial, what is the turnaround time to have the results?*

Reply: We agree with the reviewer that feasibility and turnaround time are critical for diagnostic implementation of the workflow. To better judge the potential to implement the presented workflow within a clinical study we included the turnaround time in the flowchart presented in Figure 1A. Furthermore, we include in the figure the information on points of interruption.

How many CTCs with very good genomic integrity index one needs to analyze to capture the dominant tumor clone?

Reply: This is an interesting question, because it apparently implies that we should be interested to find the predominant clone of the primary tumour. But who knows whether the predominant clone of primary tumour, which is by definition the clone most adapted to the primary site (otherwise it would not overgrow the other clones), is the clone that is seeding? Therefore, we would like to pose

the question the other way around: What is the predominant clone circulating in the blood? And then: Is that clone related or not to the predominant clone of the primary tumour?

But there are even more questions linked to it: Do CTC reflect a picture of all metastatic lesions within the body? If not, for which metastases are CTC representative? Is the detected heterogeneity in metastases (Almendro et al. Cancer Research 2014) reflected in CTC heterogeneity? Only single cell analysis will be able to study in-depth the heterogeneity of tumor cell populations and the purpose of this study is to provide a tool that enables to work on the answer for these questions within a clinical setting. However, considering the technical limitations of clinical samples, the presented workflow, for the first time, provides a solution to separate technical noise from biological implications. As summarized in Table 2, the GII score is a tool to identify WGA products of clinical single cell suitable for multiple molecular downstream analyses (targeted Sanger sequencing for PIK3CA, qPCR for ERBB2, and aCGH). Therefore, the question raised by reviewer becomes for the first time accessible by the approach presented in the manuscript.

Just one or two CTCs are enough? Or one needs to consider only cases where molecular analyses for CTCs are available for at least 5 CTCs?

Reply: We calculated the number of CTCs per patient that need to be detected by CellSearch (about 3 CTCs) that will enable performance of the molecular analyses that we presented here with at least one CTC per patient. We agree with the reviewer that the biological/medical aspect of this question needs to be analyzed, once enough cells have been isolated, analyzed and follow-up obtained. Only then we will know the meaning of any molecular finding. In our manuscript, we addressed only the technical aspect of this question, which is fundamental to answer the medical aspect.

What is the meaning of one mutated CTC among 10 wild type CTCs?

Reply: We are happy to note that our paper obviously is very thought-provocative and motivates the reviewer to ask fundamental questions regarding the biology of cancer metastasis. This is only possible if the results are technically sound. Only a good method will be helpful to address questions of this type. However, we have to stress that the purpose of our manuscript is to provide such a method and a workflow and first important insights into the wealth of biological observations that have to be studied later on.

As a first approximation to the question, the reviewer may note the observation that in many patients, especially in those with high CTC counts, therapy-relevant genetic aberrations are shared by most if not all analyzed CTCs of an individual patient. However, even in our small cohort cell-to-cell heterogeneity exists with one or few cells harboring a driver mutation, which may become relevant after therapy selection. On the other hand, in Figure 5B we now show the example of a single individual CTC lacking the characteristic *PIK3CA* M1043V mutation of the remaining CTCs. This cell displays a considerably different aCGH profile compared to the mutated sibling CTCs, (i) rendering credibility to the mutational analysis and (ii) suggesting the existence of genetically very diverse subclones.

Furthermore, it is interesting that *ERBB2* amplification is more clonal than *PIK3CA* mutation in our cohort and that *ERBB2* amplified cells show a higher number of genomic rearrangements by aCGH than *PIK3CA* mutated cells. One can only speculate if this observation is connected to higher oncogene addiction of ERBB2 positive cells compared to PIK3CA positive cells. Therefore, this issue has to be explored in more detail in the future.

For this study, we recruited a large part of the patients from the DETECT III trial. In this trial, patients with HER2-negative primary tumors are recruited and tested for CTCs by CellSearch® technology. Patients with HER2-positive CTCs are randomized between standard therapy versus standard therapy plus lapatinib. The presented approach already has been included as an amendment in the DETECT III study protocol and we hope to provide detailed answers on clinical implications of molecular single CTC analysis within the next years.

Referee #2 (Remarks):

Before replying to the many questions, we would like to thank the reviewer for his thorough analysis of our manuscript. His comments helped to improve several ambiguous aspects in our manuscript.

We did our best to address all questions with similar scrutiny and hope that the answers will satisfy the reviewer.

<u>Major comments</u>

1. *The authors make the assumption that single cell analysis is more desirable than analysis of populations of cells, and they describe their strategy as "with diagnostic intention." However, this assumption is highly debatable, especially vs. pure populations of cancer cells. Although single cell analysis may begin to reflect heterogeneity, analysis of a population provides greater statistical power and less bias. Furthermore, current tumor diagnostics, ranging from pathology/H&E, IHC, FISH, expression arrays, mutation testing etc. do not use single cell analysis for multiple reasons beyond technical limitations alone. It is in fact completely standard to evaluate tumors based on the observable phenotypes present, and to make therapeutic decisions accordingly. Thus, theoretical pros and cons of single cell assays should be discussed.*

<u>Reply:</u>. We do not *assume* that single cell analysis is more desirable. We only state the following: (i) the unit of selection in cancerous evolution is a single cell. (ii) There are clinical situations in which very few cells are the only material that can be analyzed. (iii) Since intracellular heterogeneity may be informative (see point (i)), we establish our assays with high reliability for single cells. (iv) We provide examples where single cell analysis indeed generates information not obtainable from pooled analyses. However, to meet the wish of the reviewer we included a short discussion on the need for CTC analysis.

2. *The authors' own data seems equivocal as to the utility of single cell analysis. They should more clearly indicate where single cell analysis provided more useful, clinically relevant biomarker information in this study (i.e, "with diagnostic intention").*

<u>Reply:</u> We are not sure if we understand this comment. Which data render the utility of single cell analysis equivocal? The reviewer may agree that monitoring the evolution of cancer cells may become useful for clinical decision making. Any monitoring however, will be confronted with little available material. Therefore, we do not see any point in NOT establishing such a monitoring workflow. As example for the clinical utility, we refer to our data: primary tumors may be negative for HER2 amplification or PI3K mutations, however these changes may become detectable in single CTCs during the course of disease. Detection of such therapy targets or resistance-conferring mutations may become decisive for treatment decisions.

3. *The performance characteristics of each assay tested were not well discussed. This is highly problematic since this report is primarily a feasibility study. For example, the abstract states a high number of single cells subjected to WGA, however, not all were successfully profiled. What was the success rate for each of the assay tested (i.e., sequencing, qPCR, and CGH)? What were the control quality measures for each of the assay? Please provide a section discussing performance characteristics of each assay tested (i.e., sequencing, qPCR, and CGH). Please provide a flow chart for each assay to illustrate how many sequencing, qPCR, and CGH data were excluded and how many were evaluable for the analysis.*

<u>Reply:</u> We agree with the reviewer that this point is crucial for the manuscript. Therefore, the success rates of the different molecular assays were reported in correlation to the GII score in Tables 2 and S4. To make this critical point more concise, we revised the paragraph "Genome integrity index and clinical oncogenomics" in the results section and more clearly pointed out the criteria defining performance characteristics, i.e. success or failure, of each molecular assay in the method section.

4. *The dynamic range of the log2 ratio values in CGH samples is large (Figure 2C); hence, the quality of the CGH data is in question. Also, despite the low signal-to-noise ratio, the authors*

© EMBO

were able to assess copy number calls. They should discuss clearly how the microarray data was processed and analyzed without over-fitting the data.

Reply: We published the single cell aCGH method recently in a paper (Czyz et al, 2014) that includes description of the algorithms used. Nevertheless, in the revised manuscript we provide changes in the analytical setting in the method section .

To further address the relevant question of the reviewer, we added one experiment assessing the impact of the CellSearch fixative on the experimental noise. In brief, we performed an additional aCGH experiment with WGA products of 4 untreated SKBR3 samples (3 single cells and one pool of 10 cells) isolated by manual micromanipulation and 4 SKBR3 samples treated and isolated by the presented workflow (CellSearch®/DEPArray^{TM}). As expected from the CTC/WBC data, noise (as measured by the derivative log ratio spread; DLRS) in samples generated by automated workflow is considerably higher (mean values 0.73 vs 1.12 for single cells, respectively), supporting our hypothesis on the impact of CellSearch® procedure on the DNA quality of single cells. However, genomic profiles of untreated and CellSearch® cells applying our algorithm are very similar and correspond to those of genomic DNA, although with an expected lower resolution (Figure E3).

Minor comments

Abstract

1. *Please state how many of the 510 single CTCs and 189 leukocytes WGA-amplified DNA samples were evaluable/informative for each of the assay tested (sequencing, qPCR, and/or CGH)? How many of the 66 patients had evaluable CTCs?*

Reply: We provide additional data summarizing the analysis of all cells (new Figure 2D and new Table 3) and clarified the abstract.

2. *Please clarify the statement "We defined a GII...in more than 90% of single cells". Is the claim true all for 510 single CTCs or for a smaller subset of cells belonging to GII category 3 and 4? This claim should be qualified to accurately reflect success rates.*

Reply: This success rate holds true for high quality cells only (GII 3 and 4), as shown in Table 2. We rephrased the sentence in the abstract to make this clearer.

Results

3. *(Paragraph 2, second sentence) What was the % yield (mean, median +/- s.d) after transferring enriched samples from CellSearch to DEParray cartridges?*

Reply: We added the information to the manuscript.

4. *(Paragraph 3) Figure 1C. The example provided is from a cell line. Please provide example of genotyping results from patients' CTCs.*

Reply: We are not sure that we understand the reviewer's intention. The spike-in experiment leading to the Figure 1C was performed to demonstrate single cell purity after isolation in the applied workflow. Such an experiment has to be performed with two different genotypes. How would adding patient CTCs to unrelated WBCs add any additional information? Particularly, since the ability of the method to uncover the microsatellite profile of a single diploid cell is demonstrated using single WBCs.

© EMBO

5. *(Paragraph 4) Please provide a supplementary table showing the breakdown of the 510 CTCs and 189 leukocytes WGA-amplified samples to each of the 66 patients. Were all these samples subjected to WGA QC control? What were the genome integrity indexes (GII) for each cell? Which subcategory (I-IV) does each cell belong? Please indicate what downstream molecular assay(s) was performed for each cell (sequencing, qPCR, and/or CGH). Also please indicate the HER2 status of each patient for comparison with qPCR results.*

Reply: To put ALL raw data in a table is a very unusual request. We kindly ask the **Editor** to decide whether this is needed. We already provide a very transparent overview of the data in Table 2 and new data in Figure 2D and Table 3 (see also minor point 1). In any case, these tables plus the additional all-raw-data-comprising table would be redundant and the reviewer/editor should decide which one summarizes that data best.

6. *(Paragraph 6) Please provide evidence for the statement "...WGA products comprehensively covered the single cell genome."*

Reply: By using this phrase we were referring to a result obtained during the development of the *Ampli1*™ WGA QC method. There, we showed that single cell WGA samples positive for the specific three long Mse fragments used in the QC assay in 100% showed comprehensive genomic coverage, i.e. high-quality metaphase CGH results. To avoid confusion we moved the important part of the QC development from the Expanded View to the main results additionally describing the experiments more concisely.

7. *(Paragraph 10). The relationship between the numbers mentioned in the text and those found in Figure 1D is not clear. Please clarify connection between text in the manuscript and Figure 1D.*

Reply: We thank the reviewer for the comment and agree that the mentioned part in the first manuscript version was unclear. We therefore rephrased the sentences in the revised manuscript and added Table 3.

8. (Paragraph 10) Does GII of 3 and 4 correspond to 2/3 and 3/3? Please provide consistency regarding GII nomenclature throughout the manuscript including tables and figures as well as supplementary materials.

Reply: Yes, the reviewer correctly assumes that GII 3 and 4 correspond to 2/3 and 3/3, which reflect our previous QC assay prior to the GII scoring including *KRAS* fragment. In the new version of the manuscript, we further clarified this relation.

9. (Paragraph 11). Figure 2A and 2D. The threshold for ERBB2 amplification probability score needs to be calibrated using breast cell lines with known HER2 copy number (such as those reported by your group in Czyz, PLOS ONE 2014), including MCF7 (non-amplified HER2).

Reply: Please, see accompanying confidential file summarizing results of a study on the development of the single cell qPCR and the analysis of a cohort of disseminated cancer cells of esophageal cancer patients for details on the *ERBB2* qPCR. This study is currently written up in a separate paper.

10. *(Paragraph 11). Figure 2C. Please clarify if the scale is log ratio or log2 ratio.*

Reply: We have clarified the logarithmic scale in the legend (now Figure 3E).

11. *(Paragraph 11). Figure 2E. What is the total of patients? The text mentions 42 while the table shows 40? Please clarify.*

Reply: For 2 of the analyzed 42 patients, ERBB2 status of the primary tumor was not available. To clarify, we wrote "… 8/40 (20%) of patients **with available ERBB2** status of the primary tumor…".

12. *(Paragraph 11). Figure S2. What do the gray boxes represent?*

Reply: Gray boxes reflect failure of the corresponding assay (DO = dropout). We have corrected this missing information in the revised version of the manuscript (new Figure E4).

13. *(Paragraph 12). Please elaborate on the method used for PIK3CA sequencing (Ampli1 PIK3CA Seq Kit). How were the sequencing results validated?*

Reply: Ampli1 PIK3CA Seq kit (Silicon Biosystems Spa) is based on primers to amplify an amplicon located on the corresponding Mse-fragment including the mutation hotspot on exon 9 or 20, respectively. The kit only comprises primers for the PCR amplification. We subsequently purified the PCR product and sent the sample to Sanger Sequencing (Sequiserve, Germany). We described the procedure in more detail in the new methods section.

14. *(Paragraph 13) Figure 4A. Please provide in the Discussion section a comparison of your CGH results with previous CGH data on breast CTCs (e.g. Magbanua et al 2013).*

Reply: We added a short paragraph to the discussion section. Additionally, for 4 patients we were able to perform an aCGH experiment with small pools of CTCs confirming the single cell data but also showing remarkable single cell heterogeneity (Figure 5B).

15. *(Paragraph 13) Figures S3 and S4. For consistency with Figure 2C, please superimpose the distribution of log2 ratios of probes (in genomic order) with the copy number calls (smoothed data). Please label Y-axis (log2 ratio scale).*

Reply: We followed the proposal of the reviewer, however, superimposing log2 ratios of probes with the smoothened data reduces clarity of the data shown in horizontal whole genome aberration views and we decided to stick to the original version of the figures. To make the log2 ratio spread more visible we show data for chromosome 8 and chromosome 17 comparing SKBR3 gDNA, SKBR3 single cell treated as described by Czyz et al. and SKBR3 single cells isolated and amplified by the new workflow (Figure E3).
Additionally, we thank the reviewer for noticing the missing Y-axis label (now Figures 5 and E2).

16. *(Paragraph 13) What does the phrase "genome shaping" mean? Please clarify.*

Reply: By this phrase we wanted to point out that the underlying mechanisms involved in rearrangements of chromosomes/genomes of ERBB2-amplified CTCs might be different than those of PIK3CA mutated CTCs. ERBB2-amplified show higher numbers of aberrations and are more likely to harbor additional high-level amplifications. Obviously, the type of oncogene activation has an impact on the overall genomic rearrangement.

Additional comments

17. *What are the concordance rates of replicates obtained from the same amplified DNA material? Please provide data on reproducibility of technical replicates for each of the assays: sequencing, qPCR, and CGH? Please provide correlation analysis of CGH log2 ratio data between single cells and pools of cells (if available) from the same patient.*

Reply: To address reliability of the molecular assays we show additional data. For technical replicates for aCGH we kindly refer to the study by Czyz et al. published by our group earlier this year. In this study, the high clonality of single CTCs and CTC pools (biological replicates) on a whole genome level proves reliability of the method (Figure 5B). For qPCR we show high correlation on the amplification probability of technical replicates of 27 single cell samples. Importantly, we find a very high correlation of the results in technical replicates and all samples classified as "amplified" or "non-amplified" stayed in the same category for both measurements (Figure E1). The targeted sequencing of *PIK3CA* was performed using Ampli1TM PIK3CA Seq kit for PCR amplification and subsequent Sanger sequencing by a sequencing provider (Sequiserve, Vaterstetten, Germany). For technical replicates of PCR analysis with *Ampli1*^{TM} WGA samples, we want to highlight the new results shown for the development of the QC2 multiplex assay. In the paragraph "QC2 mulitplex PCR-assay defines single cell genomic integrity index (GII)" we show a high concordance for three amplicons on long MseI fragments (1472/1521 data points, i.e. 96.8%). We want to point out that here we compared two different assays (single marker PCR QC1 and multiplex PCR QC2), with a possibly higher concordance for technical replicates with one and the same assay.

18. *How does the Ampli1 WGA method compare with other WGA protocols, e.g. WGA4 (Sigma), adapter-linker-PCR, DOP-PCR and Phi29 rolling-circle-amplification? Please provide data or discussion.*

Reply: Ampli1^{TM} WGA is the adaptor-linker method described by Klein et al (PNAS 1999 with modifications in Lancet 2002). The same WGA approach has been used for several papers on molecular single cell analysis (e.g. Schmidt-Kittler et al PNAS 2003, Schardt et al Cancer Cell 2005, Hüsemann et al Cancer Cell 2008, Stoecklein et al Cancer Cell 2008, Weckermann et al JCO 2009, Fischer et al PNAS 2013), including publications showing its suitability for aCGH analysis (Möhlendick et al PloS One 2013, Czyz et al PloS One 2014, Ulmer et al PloS Medicine 2014). Möhlendick et al directly compared the applied adaptor-linker PCR to WGA4 (Sigma) and PicoPLEX^{TM} (New England Biolabs), clearly demonstrating superiority to the first and similar results to the second single cell WGA technology. Additionally, for microdissected tissue from FFPE samples the applied WGA technology was superior to DOP-PCR (Stoecklein et al Am J Pathol 2002, Arneson et al ISRN Oncology 2012). Direct comparison between *Ampli1*^{TM} adaptor-linker PCR and MDA-based methods has not been performed to our knowledge.
Especially for the analysis of clinical samples with decreased DNA quality (e.g. due to pretreatment/fixation of the cells), as in this study, the wholly deterministic approach of the WGA method is advantageous. By digesting DNA at TTAA motif only and amplification by one universal primer, DNA fragments included in the final WGA product can be easily predicted, specific primers for downstream analysis can be designed and allelic dropout can be determined precisely (see Schardt et al Cancer Cell 2005).

To make these points clearer in the new version of the manuscript, we rephrased several sentences and added information to the discussion and methods section.

Discussion

19. *(Paragraph 3) It is unclear what the phrases "deterministic nature" of the Ampli1 WGA assay and "this disadvantage is deterministic" mean. Please explain.*

Reply: With "deterministic nature" we tried to explain the point described under point 18 above. With the second phrase "this disadvantage is deterministic" we wanted to point out, that with the

deterministic TTAA digestion as part of the *Ampli1*™ protocol one could equally predict sequences that will be more difficult to retrieve. To avoid misunderstandings we rephrased the corresponding sentences.

20. *(Paragraph 3) Please correct TTAA motive to TTAA motif.*

Reply: We thank the reviewer for noticing our spelling mistake and corrected it in the new version.

21. *(Paragraph 4) The phrase "The diagnostic relevance of assay-related single cell variation is multiplied..." is a premature assessment since the clinical relevance of single cell diagnostics is currently unclear. Please rephrase.*

Reply: We clarified the thought.

Methods

22. *Please provide a time frame for patient enrollment. How many patients were assessed, how many were evaluable?*

Reply: Please, find this information in the revised manuscript, methods section.

23. *Please explain in detail how the enriched suspension from CellSearch is counted, and then transferred and loaded to the DEParray system. What is the original volume from CellSearch? How was the volume reduced to 14uL? Please specify centrifugation parameters if performed. Please list important considerations undertaken to reduce cell loss. Also, the method cited as reference 4 in the Expanded View Results, appears to be a conference abstract only. Since this methodology is central to the present manuscript, the authors should provide a full publication and/or a detailed description in this manuscript.*

Reply: In the revised version we provided additional data and a better description in the methods section.

24. *Please provide the throughput of single cell acquisition. How many single cells can be captured in given amount of time?*

Reply: We added the information to the methods section.

25. *A published report from this group (cited as Czyz et al accepted for publication in PLOS One) was mentioned in the Expanded View section. The methods in your current work seemed to be heavily based on this report. Please discuss and cite in the main manuscript and provide an updated citation.*

Reply: We provided the correct reference.

26. *Please provide the PCR primer sequences for the GII multiplex assay.*

Reply: We added the missing information on primer sequences to the methods section.

27. *Please deposit MIAME-compliant CGH microarray data and associated clinical data/annotation to GEO or other publicly available database. The CGH data smoothing algorithm must also be made available to other researchers.*

Reply: We have deposited the data under GEO submission number GSE58192 and added this information to the manuscript. The algorithm is indicated in the methods and results section.

Referee #3 (Remarks):

In the Polzer et al paper the authors state they set to overcome 2 obstacles:

1) The isolation of individual tumor cells to purity without contaminating white blood cells (WBCs)

2) Comprehensive analysis of single cell genomes or phenotypes for diagnostic purposes

As such they provide a very useful extensive analysis and summary of breast cancer CTC evaluation which deserves publication pending some amendment and editing.

A major part of the approach is a quality control assay which they call the genome integrity index (GII) based on the detection of four specific amplified Mse I fragments. From the GII they generate a value of 0-4 which provides a measure for quality of each WGA sample generated from an isolated single cell. Although useful this is at best a guide since only 4 loci is a bit crude and subject to noise, limitations that need to be acknowledged.
They also state that they can preselect CTCs by morphology but it needs to be made clearer in the main body of the paper how morphological selection is achieved.

Reply: We are glad that the reviewer recognizes the GII as an important finding for clinical applications in single CTC molecular analysis. As we showed in Table 2, although only consisting of four genomic loci, GII highly correlates with successful molecular analysis of single cells. To further underline the importance of a quality control assay for single cell WGA, we added new paragraphs to the revised manuscript, showing the complete experimental work leading to the selection of these 4 loci and the development of *Ampli1*™ QC kit. To make the correlation between quality control assay and cell morphology clearer, we additionally rephrased this part in the revised version of the manuscript.

Specific comments:

1. *(Page 6) The authors state... "only 124/189 (65.6%) WGA libraries of single WBCs isolated from CellSearch(r)/DEPArrayTM cartridges had the same high DNA quality (chi-square P < 0.0001, Table S3). Moreover, only 191/510 (37.5%) CTCs displayed 2/3 or 3/3 QC-assay fragments (chi-square, P < 0.0001 for comparision with CellSearch-derived WBC, Table S3)."*
Comment: The authors need to more clearly define what criteria they are using for genome integrity.

Reply: We understand that the use of two different QC assays is confusing and tried to clarify this issue in the new version of the manuscript by highlighting the complete experiments leading to the development of the GII even more (see comment above). However, we feel that we still have to use both types of QC assays as we unfortunately could not test the developed GII assay on WGA-amplified samples of all 510 single CTCs and 189 leukocytes.
Based on our experience with fresh or mildly fixed single cells, in the beginning of the study we only used the three long Mse-fragments for quality control and did not expect any potentially usable amplified DNA in samples negative for all of the three specific loci. Unfortunately, during this time we put all samples without any of the long Mse-fragments (later termed GII 0 and GII 1 samples) to the waste. During the study on CellSearch® cells we were intrigued by the difference between CTCs and WBCs which lead to the development of the GII scoring system and incorporation of the short KRAS fragment to the quality control. Although it is very unfortunate that we could not test the GII

QC on all samples, we think that the number of tested single cells is still high enough and gives a good statistical correlation with successful molecular analyses (as shown in Table 2).

2. *(Page 7) The authors state..."We found CTCs from subcategory I through IV in 23 (55%), 28 (67%), 27 (64%), 19 (45%), respectively, among 42 analyzed patients. Since 33 (79%) patients harbored cells of category III or IV, which mostly display a GII of 3 or 4, it will be possible to generate high quality WGA from the majority of patients in clinical studies. "*
   Comment: Not clear - I could not understand what was said - needs clarifying.

Reply: We rephrased this paragraph to make the point clearer, restructured the text and added data in new Figure 2D and Table 3 as well as to existing Table E1.

3. *Table 2: Correlation of genome integrity index (GII) and successful performance of different molecular assays"*
   Comment: Not clear what the p values refer to.

Reply: We added an explanation in the footnotes of the table.

2nd Editorial Decision 19 September 2014

Thank you for the submission of your revised manuscript to EMBO Molecular Medicine. We apologise that it has taken longer than we would have liked to return a decision to you, also due to the holiday season and difficulties in obtaining the final evaluations

We have now received the enclosed reports from one of the Reviewers who were asked to re-assess it. In fact, Reviewer 2 did not deliver his/her report and I thus asked Reviewer 1 to assess whether you had adequately addressed Reviewer 2's comments on the first version of the manuscript. You will find both comments at the end of this letter.

I hope that the understandable inconvenience deriving from the delay, will be mitigated by the fact that the Reviewer is now globally supportive and that we will be thus able to accept your manuscript pending the following final editorial amendments:

1) For experiments involving human subjects the authors must identify the committee approving the experiments and include a statement that informed consent was obtained from all subjects and that the experiments conformed to the principles set out in the WMA Declaration of Helsinki [http://www.wma.net/en/30publications/10policies/b3/] and the NIH Belmont Report [http://ohsr.od.nih.gov/guidelines/belmont.html]. Any restrictions on the availability or on the use of human data or samples should be clearly specified in the manuscript. Any restrictions that may detract from the overall impact of a study or undermine its reproducibility will be taken into account in the editorial decision.

2) As per our Author Guidelines, the description of all reported data that includes statistical testing must state the name of the statistical test used to generate error bars and P values, the number (n) of independent experiments underlying each data point (not replicate measures of one sample), and the actual P value for each test (not merely 'significant' or 'P < 0.05'). I note that you have done so in most cases. However, rounding of the P value is acceptable only if the value is below 0,00001.

3) We are now encouraging the publication of source data, particularly for electrophoretic gels and blots (which would not apply in this case), with the aim of making primary data more accessible and transparent to the reader. Would you be willing to provide such data, if applicable? If you have any questions regarding this just contact me.

4) Every published paper now includes a 'Synopsis' to further enhance discoverability. Synopses are displayed on the journal webpage and are freely accessible to all readers. They include a short standfirst (to be written by the editor) as well as 2-5 one-sentence bullet points that summarise the

© EMBO

paper (to be written by the author). Please provide the short list of bullet points that summarise the key NEW findings. The bullet points should be designed to be complementary to the abstract - i.e. not repeat the same text. We encourage inclusion of key acronyms and quantitative information. Please use the passive voice. Please attach these in a separate file or send them by email, we will incorporate them accordingly.

Please submit your revised manuscript within two weeks at the latest. I look forward to seeing the revised final form of your manuscript as soon as possible.

***** Reviewer's comments *****

Referee #1 (Remarks):

The authors replied adequately to my comments. I would recommend for publication.

Additional remarks by Reviewer #1 on Reviewer #2's former evaluation:

I went through the reviewer 2 comments and the authors' reply. I really think that they have done a very good job. Actually the manuscript is clearly improved. Regarding the comment in the results section - paragraph 4. I would stick with the table 2 and Fig 2D in the manuscript.

© EMBO
