# Supplementary figures and images for: Molecular profiling of single circulating tumor cells with diagnostic intention
Source: EMBO Mol Med. 2014 Oct 30;6(11):1371–86. doi: 10.15252/emmm.201404033 (PMC4237466; doi:10.15252/emmm.201404033)

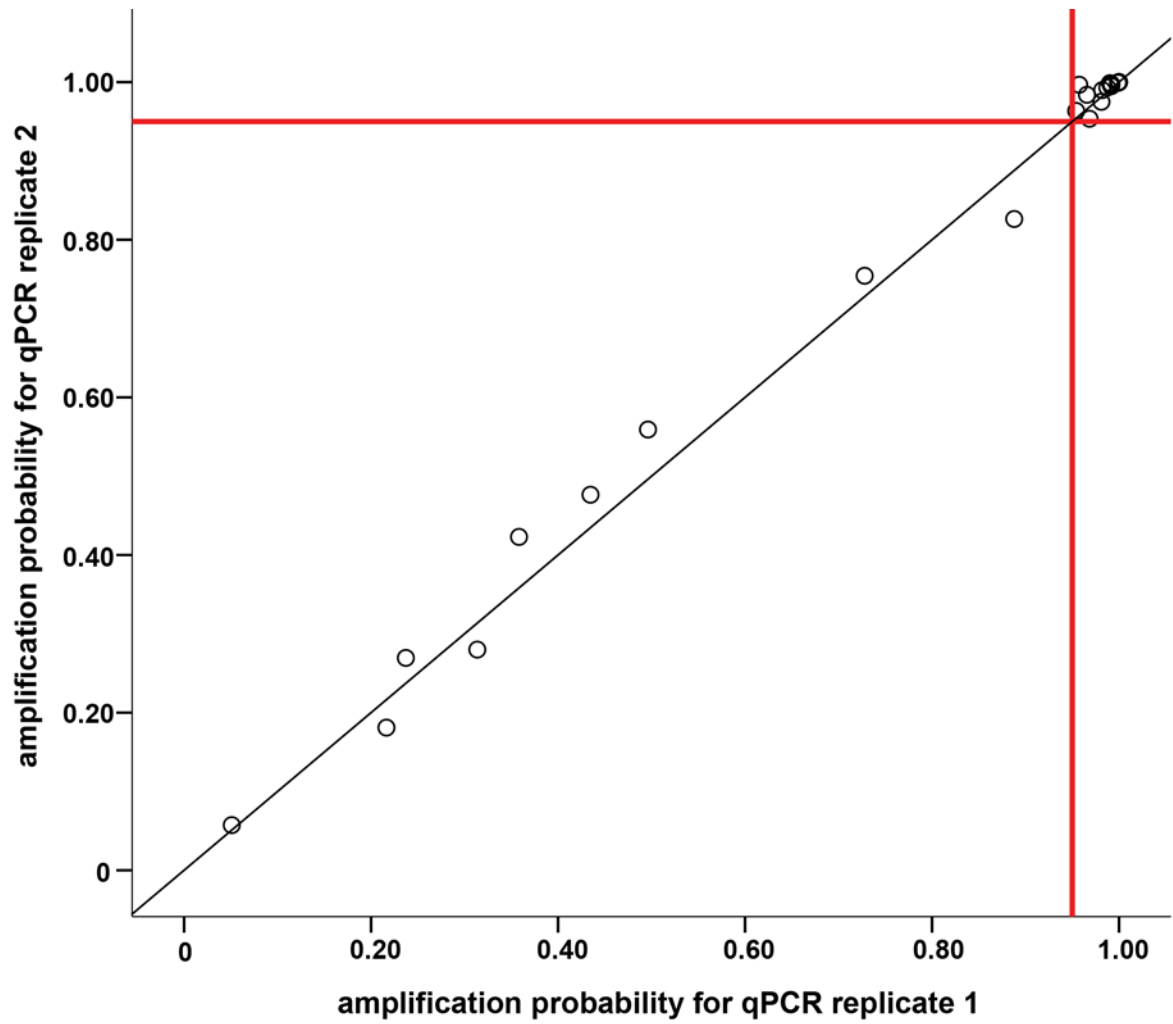

Supplement: Supplementary file 2 [file emmm0006-1371-sd2.pdf]

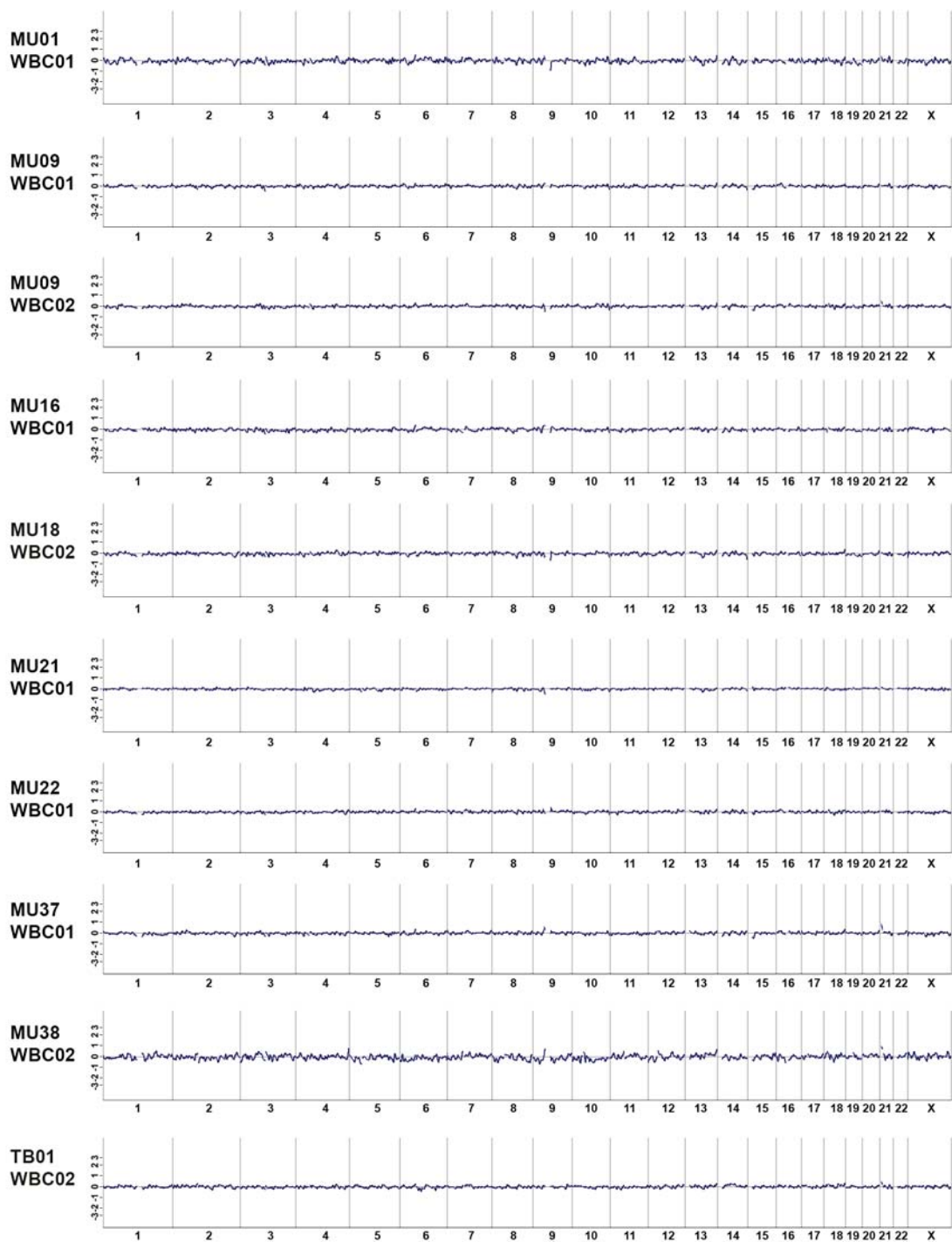

Supplement: Supplementary file 3 [file emmm0006-1371-sd3.pdf]
