# Supplementary material for: Molecular profiling of single circulating tumor cells with diagnostic intention
Source: EMBO Mol Med. 2014 Oct 30;6(11):1371–86. doi: 10.15252/emmm.201404033 (PMC4237466; doi:10.15252/emmm.201404033)
Supplement: Supplementary file 4 [file emmm0006-1371-sd4.pdf]

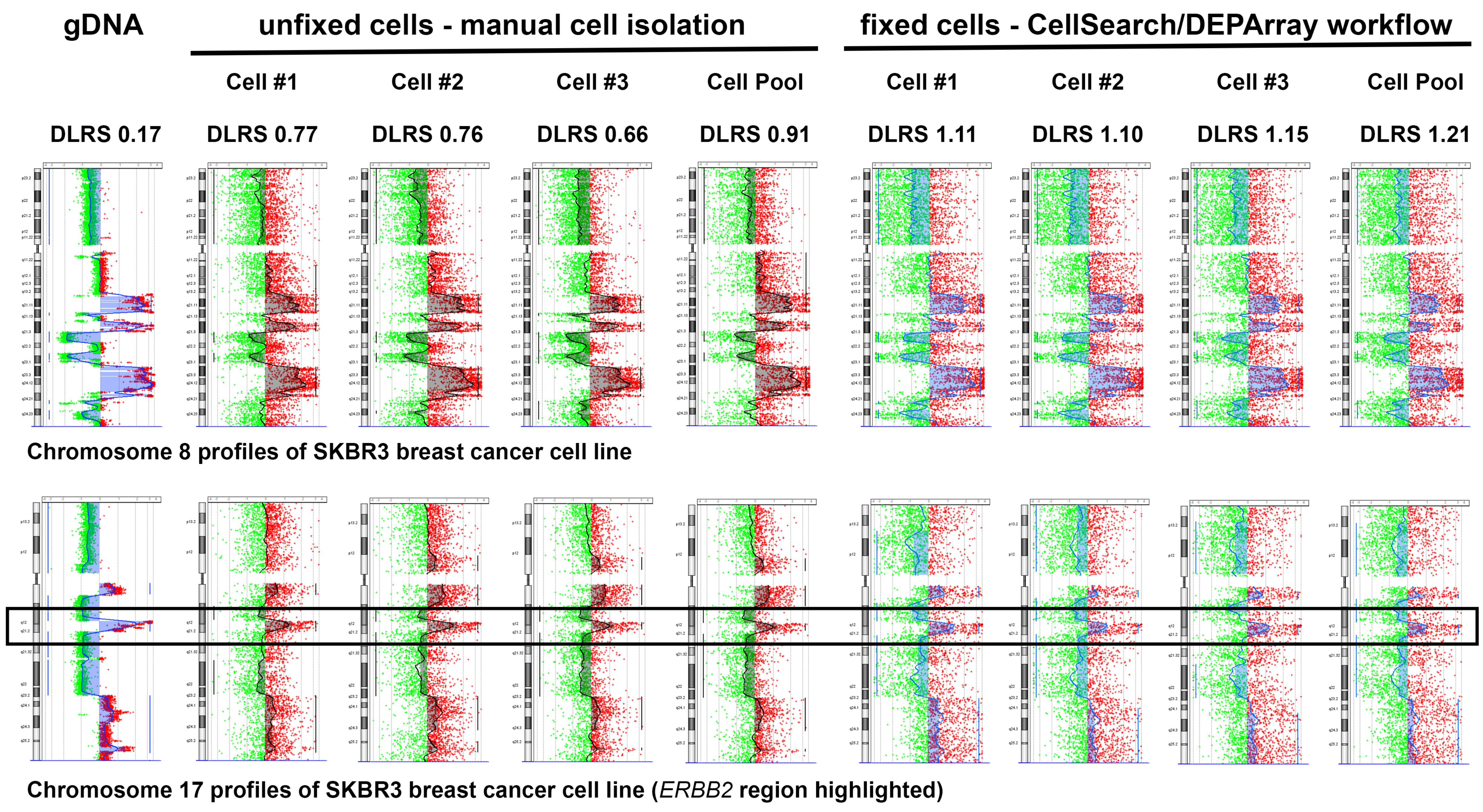
gDNA
unfixed cells - manual cell isolation
fixed cells - CellSearch/DEPArray workflow
Cell #1
Cell #2
Cell #3
Cell Pool
Cell #1
Cell #2
Cell #3
Cell Pool
DLRS 0.17
DLRS 0.77
DLRS 0.76
DLRS 0.66
DLRS 0.91
DLRS 1.11
DLRS 1.10
DLRS 1.15
DLRS 1.21
Chromosome 8 profiles of SKBR3 breast cancer cell line
Chromosome 17 profiles of SKBR3 breast cancer cell line (ERBB2 region highlighted)
