# Supplementary material for: Molecular profiling of single circulating tumor cells with diagnostic intention
Source: EMBO Mol Med. 2014 Oct 30;6(11):1371–86. doi: 10.15252/emmm.201404033 (PMC4237466; doi:10.15252/emmm.201404033)
Supplement: Supplementary file 6 [file emmm0006-1371-sd6.pdf]

CTC

IB25																																														
GII	3	4	4	4	4	4	4	4	4	4	4	4	4	4	4	4	4	4	4	4	4	4	4	3	3	3	3	3	3	3	2	4	4	4	4	4	4	4	4	3	3	2	2	1	3	3
Ex 9	wt	wt	wt	wt	wt	wt	wt	wt	wt	wt	wt	wt	wt	wt	wt	wt	wt	wt	wt	wt	wt	wt	wt	wt	wt	wt	wt	wt	wt	wt	wt	wt	wt	wt	wt	wt	wt	wt	wt	wt	wt	wt	wt	wt	DO	wt
Ex 20	mut	wt	wt	wt	wt	wt	wt	wt	wt	wt	wt	wt	wt	wt	wt	wt	wt	wt	wt	wt	wt	wt	wt	wt	wt	wt	wt	wt	wt	wt	wt	wt	wt	wt	wt	DO	wt	wt	wt	wt	DO	wt	wt	wt	wt	DO
Her2	n	n	n	n	n	n	n	n	n	n	n	n	n	n	n	n	n	n	n	n	n	n	n	n	n	n	n	n	n	n	n					n	DO	DO	DO		n	DO	DO	DO	DO	DO

MU27												
GII	4	4	4	3	3	3	2	4	3	3	4	2
Ex 9	wt	wt	wt	wt	wt	wt	wt	wt	DO	wt	wt	wt
Ex 20	mut	mut	mut	mut	mut	mut	mut	mut	mut	wt	DO	DO
Her2	n	n	n	n	n	n	n		DO	n	n	n

MU22											
GII	4	4	4	4	4	3	3	3	4	3	2
Ex 9	wt	wt	wt	wt	wt	wt	wt	wt	wt	wt	DO
Ex 20	mut	mut	mut	mut	mut	mut	mut	mut	mut	mut	mut
Her2	n	n	n	n	n	n	n	n		DO	DO

MU38										
GII	4	4	4	3	3	3	3	2	3	4
Ex 9	mut	mut	mut	mut	mut	mut	mut	mut	mut	wt
Ex 20	wt	wt	wt	wt	wt	wt	wt	wt	wt	wt
Her2	n	n	n	n	n	n	n	n		n

IB04											
GII	1	1	2	1	2	1	0	1	0	1	0
Ex 9	wt	wt	DO	wt	wt	wt	wt	DO	wt	DO	DO
Ex 20	wt	wt	wt	DO	wt	wt	wt	wt	DO	DO	DO
Her2	a	a	a	a		DO	DO	DO	DO		

IB26													
GII	4	4	4	4	3	4	0	1	0	0	0	0	0
Ex 9	wt	wt	wt	wt	wt	wt	wt	DO	DO	DO	DO	DO	DO
Ex 20	mut	mut	mut	mut	mut	mut	DO	DO	DO	DO	DO	DO	DO
Her2	n	n	n	n	n		DO						

MU09						
GII	4	4	4	3	4	2
Ex 9	wt	wt	wt	wt	wt	wt
Ex 20	mut	mut	mut	mut	wt	wt
Her2	a	a	a	a	a	a

MU35						
GII	4	4	3	3	3	2
Ex 9	mut	mut	mut	mut	DO	DO
Ex 20	wt	wt	wt	wt	wt	wt
Her2	n	n	n	n	n	n

MU20					
GII	4	4	4	3	2
Ex 9	mut	mut	mut	wt	wt
Ex 20	wt	wt	wt	wt	wt
Her2	n	n		n	n

MU18					
GII	3	2	4	4	4
Ex 9	wt	mut	wt	wt	wt
Ex 20	mut	wt	wt	wt	wt
Her2	DO		n	n	DO

MU37				
GII	4	4	4	4
Ex 9	mut	mut	wt	wt
Ex 20	wt	wt	wt	wt
Her2	a	a	a	a

MU14				
GII	4	3	2	2
Ex 9	wt	wt	wt	wt
Ex 20	mut	mut	mut	mut
Her2	n	n	n	n

MU23				
GII	4	4	4	3
Ex 9	mut	mut	wt	DO
Ex 20	wt	wt	wt	DO
Her2	n	DO	n	n

MU21			
GII	4	4	2
Ex 9	wt	wt	wt
Ex 20	wt	wt	wt
Her2	a	a	a

IB14				
GII	4	3	0	0
Ex 9	wt	wt	wt	DO
Ex 20	mut	wt	wt	DO
Her2	n		DO	

MU35			
GII	3	4	3
Ex 9	mut	DO	DO
Ex 20	wt	wt	wt
Her2	n	n	n

TB04					
GII	3	4	4	2	0
Ex 9	wt	wt	wt		
Ex 20	wt	wt	wt		
Her2	a	n	n	DO	DO
