# Supplementary material for: Molecular profiling of single circulating tumor cells with diagnostic intention
Source: EMBO Mol Med. 2014 Oct 30;6(11):1371–86. doi: 10.15252/emmm.201404033 (PMC4237466; doi:10.15252/emmm.201404033)
Supplement: Supplementary file 7 [file emmm0006-1371-sd7.pdf]

Table S2. Primer and amplicon information for the QC1 and QC2 assays.

Oligonucleotide ID	Chromosomal location	Length of Mse fragment	Sequence	Annealing temperature	Length of PCR fragment	Included in assay
BCR-TT forward	22q	1936 bp	CGTGGACAACTACGGAGTTG	61°C	323 bp	
BCR-TT reverse			TCAGCCTCAGGACTCTTGTG			
D5S2117 forward	5q	1376 bp	CCAGGTGAGAACCTAGTCAG	58°C	140 bp*	QC1, QC2
D5S2117 reverse			ACTGAGTCCTCCAACCATGG			
TP53 Ex2/3 forward	17p	1374 bp	GAAGCGTCTCATGCTGGATC	58°C	301 bp	QC1, QC2
TP53 Ex2/3 reverse			CAGCCCAACCCTTGTCCTTA			
CK19 forward	6q	1146 bp	GAAGATCCGCGACTGGTAC	58°C	614 bp	QC1, QC2
CK19 reverse			TTCATGCTCAGCTGTGACTG			
IGF2R forward	6q	647 bp	GCCACTGTCGAAGTCTGCA	58°C	217 bp	
IGF2R reverse			GGATCTTGGTACCACTCATG			
RUFY2 forward	10q	458 bp	GTTGAGGGCTTCATCAACACCCA	64°C	104 bp	
RUFY2 reverse			CAGCTAGGAACTCCAGGAATCA			
SMYD1 forward	2p	287 bp	GGGTGACCTGCTTGACATC	55°C	163 bp	
SMYD1 reverse			CTTTTCCCTGAAGGTCTTAG			
PHAGTR2 forward	6q	239 bp	ACTGAACAGAGCAGGTCTAC	58°C	205 bp	
PHAGTR2 reverse			TGTGAGAAAGACTTGGAGTT			
KRAS forward	12p	192 bp	ATAAGGCCTGCTGAAAATGAC	58°C	91 bp	QC2
KRAS reverse			CTGAATTAGCTGTATCGTCAAGG			

* microsatellite marker with varying sequence length; homozygous samples show one band, heterozygous samples two bands in the size range 108-166 bp
