# Supplementary material for: Molecular profiling of single circulating tumor cells with diagnostic intention
Source: EMBO Mol Med. 2014 Oct 30;6(11):1371–86. doi: 10.15252/emmm.201404033 (PMC4237466; doi:10.15252/emmm.201404033)
Supplement: Supplementary file 9 [file emmm0006-1371-sd9.pdf]

Table S3. QC-1 assay: Detection of long Mse-fragments after *Ampli*1™ WGA

		Detected sequences by QC1-assay (3 markers)			
	**n**	**3/3**	**2/3**	**1/3**	**0/3**
**Single WBCs (unfixed)**	88	80 (90.9%)	3 (3.41%)	0	5 (5.68%)
**Single WBCs (CellSearch®)**	189	73 (38.6%)	51 (27.0%)	20 (10.6%)	45 (23.8%)
**Single CTCs (CellSearch®)**	510	102 (20.0%)	89 (17.5%)	55 (10.8%)	264 (51.8%)
