# Supplementary material for: Molecular profiling of single circulating tumor cells with diagnostic intention
Source: EMBO Mol Med. 2014 Oct 30;6(11):1371–86. doi: 10.15252/emmm.201404033 (PMC4237466; doi:10.15252/emmm.201404033)
Supplement: Supplementary file 10 [file emmm0006-1371-sd10.pdf]

Table S5. Genomic integrity index predicts downstream molecular analyses in single CTCs and WBCs.

Sample	Molecular assay	n	GII 0*	GII 1*	GII 2*	GII 3*	GII 4*	P Value§
**CTCs**	**PIK3CA Exon 9**	261	5/20 (25.0%)	12/20 (60.0%)	36/48 (75.0%)	67/77 (87.0%)	93/96 (96.9%)	<0.00001
	**PIK3CA Exon 20**	261	7/20 (35.0%)	16/20 (80.0%)	41/48 (85.4%)	73/77 (94.8%)	93/96 (96.9%)	<0.00001
	**PIK3CA complete**	261	3/20 (15.0%)	11/20 (55.0%)	33/48 (68.8%)	64/77 (83.1%)	91/96 (94.8%)	<0.00001
	**ERBB2 qPCR**	246	3/11 (27.3%)	8/16 (50.0%)	28/47 (59.6%)	65/77 (84.4%)	88/95 (92.6%)	<0.00001
	**aCGH**	40	not assessed	not assessed	3/4 (75.0%)	6/8 (75.0%)	28/28 (100%)	0.023
**WBCs**	**PIK3CA Exon 9**	122	2/3 (66.7%)	2/5 (40.0%)	12/14 (85.7%)	35/40 (87.5%)	53/60 (88.3%)	0.048
	**PIK3CA Exon 20**	122	1/3 (33.3%)	2/5 (40.0%)	14/14 (100%)	36/40 (90.0%)	56/60 (93.3%)	0.0003
	**PIK3CA complete**	122	1/3 (33.3%)	1/5 (20.0%)	12/14 (85.7%)	33/40 (82.5%)	50/60 (83.3%)	0.0004
	**ERBB2 qPCR**	105	0/1	0/2	13/14 (92.9%)	30/35 (85.7%)	48/53 (90.6%)	0.00036
	**aCGH**	10	not assessed	not assessed	1/1 (100.0%)	1/1 (100.0%)	8/8 (100%)	not assessed

* GII = Genome integrity index; GII 0: no fragment detected by QC2-assay; GII 1: QC2-assay negative for the three long markers, but KRAS fragment detected; GII 2: 1/3 long MseI fragments of QC2-assay detected; GII 3: 2/3 long MseI fragments of QC2-assay detected; GII 4: 3/3 long MseI fragments of QC2-assay detected

§ Tested by Chi-square
