# Supplementary material for: Molecular profiling of single circulating tumor cells with diagnostic intention
Source: EMBO Mol Med. 2014 Oct 30;6(11):1371–86. doi: 10.15252/emmm.201404033 (PMC4237466; doi:10.15252/emmm.201404033)
Supplement: Supplementary file 11 [file emmm0006-1371-sd11.pdf]

Table S6. PIK3CA mutations in breast cancer CTCs

patient ID*	Stage	detected mutation	number of wildtype CTC§	number of mutated CTC	mutational status CTC pool
MU01	Stage IIA	E542K, exon 9	1	1	not tested
MU09	Stage IV	H1047R, exon 20	2	4	not tested
MU14	Stage IV	H1047L, exon 20	0	4	not tested
MU16	Stage IV	H1047R, exon 20	1	1	not tested
MU18	Stage IV	H1047R, exon 20	3	1	E545K, exon 9
MU20	Stage IV	E545K, exon 9	2	2	E545K, exon 9
MU22	unknown	H1047R, exon 20	0	10	H1047R, exon 20
MU23	Stage IV	E545K, exon 9	1	2	not tested
MU27	Stage IV	M1043V, exon 20	2	8	M1043V, exon 20
MU31¶	Stage IV	H1047R, exon 20	0	1	not tested
MU35	Stage IV	E542K, exon 9	0	5	not tested
MU37	Stage IV	E542K, exon 9	2	2	not tested
MU38	Stage IV	E542K, exon 9	1	8	E542K, exon 9
TB07	Stage IV	E545K, exon 9	0	1	not tested
IB14	Stage IV	F1039S, exon 20	6	1	Wildtype
IB25	Stage IV	W1051*, exon 20	37	1	Wildtype
IB26	Stage IV	H1047R, exon 20	0	5	H1047R, exon 20

* patient ID indicates the clinical sample that acquired the sample (MU = Munich, TB = Tuebingen, IB = Milano)

§ confirmed wildtype status for the mutated exon of the patient (exon 9 or 20, respectively), cells with dropout for the exon of interest were not listed. Importantly, all three single CTC of patient MU18 labeled wildtype have confirmed wildtype status for both exon 9 and 20.

¶ patient MU31 is not included among the 16 patients mentioned in the main text, as we were not able to obtain sequence data for both *PIK3CA* exons
