# Supplementary material for: Molecular profiling of single circulating tumor cells with diagnostic intention
Source: EMBO Mol Med. 2014 Oct 30;6(11):1371–86. doi: 10.15252/emmm.201404033 (PMC4237466; doi:10.15252/emmm.201404033)
Supplement: Supplementary file 13 [file emmm0006-1371-sd13.pdf]

Table S7. qPCR primers.

Primer		Sequence	Annealing temperature
HER2-310	forward	CTGAGGTGGGAGACTTGCT	64°C
	reverse	ACTCGATGCTTGCTACTGCA	
HER2-315	forward	GACAAAATGTAGAGCCAGACCATTTGG	64°C
	reverse	GGATCAGCACTGTGAGATTC	
HER2-E	forward	TGGCACAGTGAAGCACGGGAAAG	66°C
	reverse	GGCCCTCCTCCAAGCTTGATCCT	
AGTR1	forward	CAGGAGATGAGAGTTCCAGA	55°C
	reverse	GTACCAGGTGCAAGTGTAGC	
OPN1LW	forward	CTGTGCTTACCAAAGGCTTC	60°C
	reverse	GACTCTACCCAGGTTTCTAG	
CACNA1D	forward	GGTGGCTGTACGGGTATATT	55°C
	reverse	CCTGTGAAGTTTCAGGCTGTC	
GZMB	forward	AGACTATGCACCTGCCTGGA	55°C
	reverse	GCCTCCTGGTAAGTTTGCAG	
RUFY2	forward	GTTGAGGGCTTCATCAACACCCA	64°C
	reverse	CAGCTAGGAACTCCAGGAATCA	
SMYD1	forward	GGGTGACCTGCTTGACAT	55°C
	reverse	GGGTGACCTGCTTGACAT	
