# Supplementary material for: Molecular profiling of single circulating tumor cells with diagnostic intention
Source: EMBO Mol Med. 2014 Oct 30;6(11):1371–86. doi: 10.15252/emmm.201404033 (PMC4237466; doi:10.15252/emmm.201404033)
Supplement: Supplementary file 14 [file emmm0006-1371-sd14.pdf]

SUPPLEMENTARY FIGURE LEGENDS

Figure S1. Technical replication of *ERBB2* qPCR measurements.

Repeat experiment of 27 single cell WGA libraries by single cell qPCR assay. In all cases, samples were correctly classified as "amplified" or "non-amplified" based on the calculation of the *ERRB2* amplification probability score (red lines indicate 0.95 threshold). Correlation between amplification probability scores of the two replicates was $r = 0.98$ (Spearman-Rho, $P < 0.00001$).

Figure S2. Genomic profiles of single white blood cells.

Genomic overview of 10 isolated single WBCs of 9 breast cancer patients after hybridization on Agilent CGH microarrays shows balanced profile fo all cells (chromosomes 1 to Y on the x-axis, y-axis is shown in log 2 ratio scale).

Figure S3. Influence of CellSearch® / DEPArray™ workflow on single cell aCGH

WGA products of four untreated SKBR3 samples (three single cells and one pool of 10 cells) isolated by manual micromanipulation and four SKBR3 samples treated and isolated by the presented workflow (CellSearch®/DEPArray™) were hybridized on CGH arrays. Log2 ratios of hybridization signals for all probes on the array are depicted by red (deviation to the right of 0) and green dots (deviation to the left of 0). Hybridization noise (as measured by the derivative log ratio spread; DLRS) in samples generated by the automated workflow was considerably higher than for unfixed cells. However, aberration calls are highly similar (as indicated by the dark blue lines along) and correspond to those of genomic DNA (profile on the far left).

Figure S4. Molecular heterogeneity for *ERBB2* and *PIK3CA* in breast cancer CTCs of individual patients.

Mutation analysis of PIK3CA and amplification status for ERBB2 for CTCs from all patients with at least 3 recoveries of CTCs (columns) that could be evaluated and that harbor at least one alteration in one of the recoveries. Recoveries with a pool of two or more cells are indicated by a black bar above

the recovery data. Pools were not assessed for *ERBB2* amplification (white squares). Red: point mutations (mut) in *PIK3CA* hot-spots or *ERBB2* amplification (a). Green: wild-type (wt) *PIK3CA* sequence or no amplification (n) for *ERBB2*. Gray: analysis drop-out (DO). All recoveries are displayed left to right by decreasing ‘tumor burden’ (i.e. # of mutations found), then by decreasing ’data score’ (i.e. number of data points for molecular characterization).

Note that for the same patient sampling, heterogeneity is often found at single-cell level: i) the same mutation or gene amplification is found along with wild-type or normal copy number, e.g. MU20 ii) two different mutations of *PIK3CA* are found in different CTCs, e.g. MU18, iii) among CTCs with *ERBB2* amplification some have an additional *PIK3CA* mutation that other CTCs do not have, e.g. MU09, MU37.
